# Supplementary material for: Observing ground-state properties of the Fermi-Hubbard model using a scalable algorithm on a quantum computer
Source: Nat Commun. 2022 Oct 11;13:5743. doi: 10.1038/s41467-022-33335-4 (PMC9553922; doi:10.1038/s41467-022-33335-4)
Supplement: Supplementary file 1 — Supplementary Information [file 41467_2022_33335_MOESM1_ESM.pdf]

Supplementary Information for "Observing ground-state properties of the Fermi-Hubbard model using a scalable algorithm on a quantum computer"

Stasja Stanisic,[1] Jan Lukas Bosse,[1,2] Filippo Maria Gambetta,[1] Raul A. Santos,[3]
Wojciech Mruczkiewicz,[4] Thomas E. O'Brien,[4] Eric Ostby,[4] and Ashley Montanaro[1,2]
[1]*Phasecraft Ltd., Bristol, UK*
[2]*School of Mathematics, University of Bristol, UK*
[3]*Phasecraft Ltd., London, UK*
[4]*Google Quantum AI, Mountain View, CA, USA*
(Dated: September 8, 2022)

Supplementary Note 1 – Physics of the Fermi-Hubbard model

The Fermi-Hubbard model[1] encapsulates the effect of inter-electron interactions in a single narrow band system. It was proposed to describe d-orbitals in transition metals. In these systems, the strength of the interactions between localised electron orbitals is comparable with the bandwidth. This makes it a natural candidate for studying strongly correlated effects in solids[2]. The phenomenology of different cuprates that develop high-temperature superconductivity (e.g. $\mathrm{Sr_{1-x}Ca_xCuO_2}$) has been linked with the different phases of the model, although is still open if the model itself can support a high temperature superconducting phase[3].

A general tight-binding description of the electron Hamiltonian in solids is

$$H = -\sum_{\alpha\beta} t_{\alpha\beta} a^\dagger_\alpha a_\beta + \sum_{\alpha\beta\gamma\delta} U_{\alpha\beta\gamma\delta} a^\dagger_\alpha a^\dagger_\beta a_\gamma a_\delta, \tag{1}$$

where $t_{\alpha\beta}$ contains the contributions from the kinetic energy and ion potential of the lattice, while $U_{\alpha\beta\gamma\delta}$ parameterizes the electron-electron interactions (here $\alpha, \beta, \gamma, \delta$ contain all the labels of the fermion operator). In the atomic limit, where the overlap between orbitals at different sites decays exponentially, the leading contribution of the interaction term comes from density-density coupling. In the atomic limit of the 1-band case without spin-orbit coupling, the tight-binding description becomes

$$H = -\sum_{ij} t_{ij,\sigma} a^\dagger_{i,\sigma} a_{j,\sigma} + U \sum_i n_{i,\uparrow} n_{i,\downarrow}, \tag{2}$$

where $n_{i,\sigma} = a^\dagger_{i,\sigma} a_{i,\sigma}$. For homogeneous nearest neighbour hopping $t_{ij} = t$ for adjacent sites i, j. and measuring energies in units of t, this Hamiltonian becomes Eq. (1) in the main text.

In 1D, the Fermi-Hubbard model is solvable by the Bethe ansatz, meaning that by solving the Bethe equations an efficient description of the energy and ground state exists[4]. In 1D this model shows spin-charge separation, as its quasiparticles are spinons and holons. The system belongs to the universality class of the Luttinger liquid[5], away from half-filling. Exactly at half-filling, the system becomes a Mott insulator, developing a finite gap to addition of particles. In general, at zero temperature, at least two transitions are expected for dimensions larger than one. For small fillings, the encounter probability of two particles is small, making the interaction unimportant and the system metallic (although interactions could still affect the anomalous exponents of different correlators). At half-filling there is one electron per site on average and at large enough interaction strength U it is expected that the system adopts the configuration of exactly one electron per site. This state is the Mott insulator. At larger fillings the system should again become metallic, where the carriers are holes. In general, two transitions are expected as a function of density, from metallic to the Mott insulator near half-filling, and back to metallic at higher fillings. In 1D this happens at exactly half-filling for $U > 0$. In higher dimensions the location of these transitions is still unresolved. The nature of the Mott transition is a matter of debate, where different mechanisms are expected to contribute, from the localization of quasiparticles[6] to the development of magnetic instabilities[7].

Supplementary Note 2 – Prior work

Experimental implementations of quantum algorithms for the Fermi-Hubbard model. The Fermi-Hubbard model has long been proposed as a plausible application of quantum computing. However, to our knowledge, there has been no experimental demonstration of finding the ground state of instances of the model using a quantum computer without introducing some notion of compression or restriction to a subspace.

Linke et al.[8] found the ground state of the 1×2 Fermi-Hubbard model using a discretised adiabatic algorithm implemented in an ion trap experiment. Using symmetries of the system, this problem can be mapped to 2 qubits. Linke et al. produced two copies of the ground state and used these to measure its entanglement as measured by Rényi entropy, via a controlled-swap gate. The overall circuit uses 5 qubits and 31 2-qubit gates (12 to produce each copy of the ground state, and 7 for the controlled-swap).

Montanaro and Stanisic[9] demonstrated the VQE al-

gorithm for the Fermi-Hubbard model, again on a 1×2 system compressed to 2 qubits and using 2 2-qubit gates, in superconducting qubit hardware. Suchsland et al.[10] used symmetries to compress a 4-site Hubbard ring at half-filling to 4 qubits, and applied the VQE algorithm on a different superconducting qubit platform to find the ground state. Their variational ansatz used 3 2-qubit gates.

Very recently, Gard and Meier[11] proposed the use of VQE for finding the ground state of the (noninteracting) single-particle 1D Fermi-Hubbard model, which can be solved efficiently classically for any size, as a benchmark for quantum computers. These authors used classical simulations of VQE to find the optimal parameters, and then executed the corresponding circuits, with up to 21 2-qubit gates, to measure the energy on a variety of quantum hardware platforms.

Outside of the VQE paradigm, a closely related work implemented a simulation of time-dynamics of the Fermi-Hubbard model[12], starting with a ground state of the noninteracting model prepared by Givens rotations, and time-evolving according to a Trotterised version of the 1×8 Fermi-Hubbard Hamiltonian with occupation number 4 or 6. That work was able to demonstrate separation of spin and charge dynamics; meaningful results were obtained for circuits of 2-qubit gate depth over 400. Different error-mitigation techniques were used in that work to those employed here: averaging over different choices of qubits, Floquet calibration, and a rescaling method. Other contrasts are that the present work includes the optimisation component of VQE, considers a $2 \times L_y$ system, and computes many different observables.

Other large-scale implementations of variational algorithms. Beyond Fermi-Hubbard, in related work VQE has been demonstrated in the context of quantum circuits for preparing Hartree-Fock states on up to 12 qubits with high accuracy on a Google Sycamore processor[13]. These states can be efficiently prepared via Givens rotations in a similar way to the initial state used in our VQE experiment. The VQE procedure is therefore solely used to correct for errors in these Givens rotations. As Hartree-Fock states are efficiently simulable classically, algorithms creating them are excellent benchmarks of quantum computer performance, but cannot achieve exponential speedups over classical computation.

VQE has been applied to a number of other systems in quantum chemistry. The largest such experiment that we are aware of applied a hardware-efficient ansatz combined with error-mitigation techniques to find ground states of H_2 and LiH, using up to 6 qubits and 2-qubit gate depth 3[14]. VQE has also been used to demonstrate the metal-insulator transition in H_3, via an experiment with 3 qubits and 2 2-qubit gates[15]. See Ref. 16 for a survey of many other small implementations up to 2018.

Another domain where a variational approach can be used is quantum algorithms for optimisation, via the Quantum Approximate Optimisation Algorithm[17] (QAOA). In QAOA, one aims to find good approximate solutions to hard constraint satisfaction problems which can be expressed as finding the ground state of a classical Hamiltonian. The QAOA algorithm optimises over parametrised quantum circuits where the elementary operations are time-evolution according to the terms in this Hamiltonian, and time-evolution according to a transverse "mixer" term. As QAOA is solving a problem where the goal is to output a classical bit-string, rather than (for example) to output an accurate estimate of an energy, it is substantially easier to obtain meaningful results even in the presence of high levels of noise; even if the success probability is exponentially small in the problem size, this can still be distinguishable from random noise, given enough samples. The largest QAOA experiment that we are aware of used up to 23 qubits on a Google Sycamore processor and up to 3 variational layers[18].

Numerical studies. The variational ansatz we used in our experiments is based on one developed and validated in previous work, which simulated the VQE algorithm classically for Fermi-Hubbard instances on up to 12 sites[19]. Independent work[20] has very recently used classical simulation to study the ability of a low-depth variational ansatz to represent features of the Fermi-Hubbard model such as spin correlations, double occupancies, energy and ground-state fidelity (energies and fidelities were already computed for $U = 2$ in Ref. 19). The results presented are for 1D chains on 8 sites.

Other experimental implementations of the Fermi-Hubbard model. In recent years, many fundamental contributions to the study of the Fermi-Hubbard model have been obtained within special-purpose analogue quantum simulators[21–24]. Among these, cold atoms in optical lattices play a central role thanks to the high degree of control of system parameters, such as dimensionality, hopping, and interaction strength, and single-atom resolution imaging techniques[21,24–27]. These systems provide access to a plethora of observables which are challenging to measure in solid state devices, such as full counting statistics of local degrees of freedom and higher-order correlation functions.

Thanks to these features, these experiments shed light on the interplay between doping and magnetism in low-temperature Fermi-Hubbard systems in regimes which are difficult to explore even with state-of-the-art numerical techniques. For instance, in 1D Fermi-Hubbard chains the possibility to measure non-local correlation functions in real space allowed the demonstration of spin-charge separation[28] and probing the onset of incommensurate magnetism[29]. On the other hand, experiments on doped 2D Fermi-Hubbard models explored the effects of doping on the long-range antiferromagnetic order[30], studied the crossover from a polaron metal to a Fermi liquid[31], and unveiled exotic transport properties[32,33].

Despite the many groundbreaking insights provided by cold atoms in recent years, simulation of condensed-matter models with analogue quantum simulators has to face two main challenges[26]. First, the temperatures that

can be achieved in current setups are usually too high to reach zero-temperature ordered phases. And second, to explore the onset of long-range order, it is essential to realize larger systems with the same degree of single-atom and single-site control. Digital quantum simulators have the potential to attain zero-temperature phases in a more efficient way, allow full control of each individual parameter of the model, such as coupling strengths and local fields, and eventually should enable access to larger and more general systems.

Supplementary Note 3 – Complexity and scaling of the Efficient Hamiltonian Variational ansatz

Quantum circuit complexity. The complexities of the largest circuits that we executed are summarised in Supplementary Table I. It is interesting to note that for a 2×4 lattice, the most complex instances in terms of circuit complexity were not at half-filling; this is because in this specific case, one fewer layer of Givens rotations is present than worst-case bounds would suggest[34].

Scaling of algorithms using rectangular and zig-zag configurations. The quantum circuit depth for each layer of the EHV ansatz for a $L_x \times L_y$ lattice, $L_x \leq L_y$, with no restrictions on quantum circuit topology[19], and assuming that an arbitrary 2-qubit operation can be implemented with one hardware-native gate, is as low as $2L_x + 1$ (for even L_x). Almost all gates that occur in the algorithm act across nearest neighbours in the Jordan-Wigner line, with the exception of onsite gates and basis transformations necessary for measuring vertical hopping terms.

To implement this circuit using a $L_x L_y \times 2$ rectangular configuration of qubits on a device, we can associate one row with each spin-type, and associate modes within each spin sector with qubits in the Jordan-Wigner ordering (see Ref. 35 for a related proposal). Then onsite gates are local, so there is no additional cost per ansatz layer, and the only remaining long-range transformation is the basis transformations for measuring vertical hopping terms.

To implement these, it is sufficient to reorder a pair of rows in the Fermi-Hubbard lattice such that vertically neighbouring pairs become horizontally neighbouring. It is easiest to illustrate a procedure for this with an example. If we label modes in the first row of a $4 \times L_y$ lattice as $1, 2, 3, 4$, and modes in the second row as A, B, C, D, we want to transform from the ordering $1234ABCD$ to the ordering $1D2C3B4A$. This transformation can be split into two parts: flipping $ABCD$ to $DCBA$, and then transforming $1234DCBA$ to $1D2C3B4A$. For rows of length L_x the first part can be implemented with L_x layers of FSWAP operations in an alternating even-odd pattern[36]. The second part requires $L_x - 1$ layers of FSWAPs, in a triangle configuration beginning at the middle, to interleave the first and second rows. The overall cost is therefore $2L_x - 1$ layers of FSWAP operations; for the special case of a $2 \times L_y$ lattice, we can improve this to just 1 layer (Fig. 1 in the main text).

Lattice	N_{occ}	Embedding	Circuit depth	2-qubit depth	2-qubit gates
1×4	4	Rectangle	41	20	64
1×8	8	Zig-zag	53	26	140
2×4	7	Zig-zag	65	32	176

Supplementary Table I. Largest circuit complexities for the configurations considered in our experiments. The 1×4 experiments used a depth 2 VQE ansatz, while the other experiments used a depth 1 ansatz. Circuit complexities depend on which energy measurement is being performed in the VQE algorithm; stated complexities are the maximal ones over these circuits, showing the occupation numbers where these are achieved.

All of the same arguments hold for the zig-zag configuration that we use in our experiments, except that now we need an additional layer of FSWAP gates before and after the onsite gates, giving an overall cost of $2L_x + 3$ 2-qubit gate depth per ansatz layer. We remark that previous work giving complexity bounds for nearest-neighbour and Sycamore architectures[19] considered more "balanced" configurations suitable for fitting more modes into a small quantum processor whose width and height are closer in size; this led to larger bounds ($4L_x + 1$ for nearest-neighbour and $6L_x + 1$ for Sycamore, respectively).

We remark that instances of the Hamiltonian Variational ansatz we use with one variational layer can be simulated efficiently classically[37,38], so in order to use our approach to demonstrate a significant quantum speedup for Fermi-Hubbard instances beyond sizes accessible to classical exact diagonalisation, higher-depth variational circuits must be employed.

Supplementary Note 4 – Error mitigation

The error mitigation techniques we used can be divided into three categories: low-level techniques which are not specific to the Fermi-Hubbard model and are targeted at the particular hardware platform used; techniques based on the symmetries of the Fermi-Hubbard model; and a technique that is designed to mitigate errors in fermionic simulation algorithms. For a survey of other error mitigation techniques, see Ref. 39.

1. Low-level error mitigation

Circuit structure. We ensure that our quantum circuits are of a form where we alternate between layers of single-qubit and two-qubit gates. This is similar to the circuit topology used for quantum supremacy experiments[40], and is advantageous because two-qubit gates generally take longer to execute than single-qubit

gates, and the time taken to execute a layer is equal to the time for the slowest gate in it.

Qubit selection. Our experiments use a set of up to 16 qubits in a particular orientation. On the quantum processor we used, there were four such sets of 16 qubits available, and the choice of which set to use could make a significant difference to experimental performance. In a VQE experiment, a straightforward metric to use to select qubits is the energy measured for some choice of parameters. In general, measuring lower energies is better, as we expect decoherence to increase the energy; coherent errors will also usually increase the energy, if the initial state's energy is close to the lowest possible within that VQE ansatz. We therefore selected qubits for our experimental run by choosing fixed parameters (all zeroes), measuring the energy at these parameters on all four sets of possible qubits, and choosing the qubits which achieved the lowest energy. Spot checks throughout the experimental run showed that this set of qubits remained high-quality. Unlike some previous work[12], we found that averaging results over different qubit sets was not advantageous in reducing the final error (even when other error-mitigation techniques were also applied); it was usually better just to pick one "best" set of qubits and use them throughout.

Run selection. For each set of VQE parameters, we carried out 3 experiments at different times to create the corresponding VQE state and generate energy estimates. We then selected the run which returned the lowest "raw" energy estimate (following postselection by occupation number (see below)) for subsequent calculations. The intent behind this is that we expect the level of noise and errors experienced by the qubits at a particular time to be correlated with the measured energy, so a lower energy should correspond to a higher-accuracy experiment.

Spin echo. For our experiments on 16 qubits we used a technique inspired by the concept of refocusing by spin echo in NMR[41]. Given an unknown unitary operation $U = e^{i\theta Z}$, then as $XUX = U^{-1}$, implementing the sequence $UXUX$ produces the identity map. If we think of U as an error whose precise form is unknown, this allows the error to be corrected. Here we implemented this idea by sandwiching alternating layers of 2-qubit gates with layers of X gates (Supplementary Figure 1). As $X^{\otimes 2}$ commutes with the $\sqrt{\text{iSWAP}}$ gate, on a perfect quantum computer this would leave the unitary operation implemented unchanged. On an imperfect quantum computer, it may help to correct errors. Introduction of X gates was previously found to reduce errors on an idle qubit in a similar superconducting quantum processor[42], and more complex dynamical decoupling sequences[43] have been demonstrated to significantly reduce decoherence in other superconducting quantum computing systems[44].

In our experiments the effect on errors was substantial, whichever subset of qubits was used. As well as correcting unwanted Z rotations, another possible explanation for this effect may be that 2-qubit gates are known to experience a substantial "parasitic CPHASE[12]", manifesting as an undesired phase acting on $|11\rangle$. The X gates move this phase to $|00\rangle$, which may reduce its effect over the circuit as a whole. Also observe from Supplementary Figure 1 the substantial variations in measured energies over time.

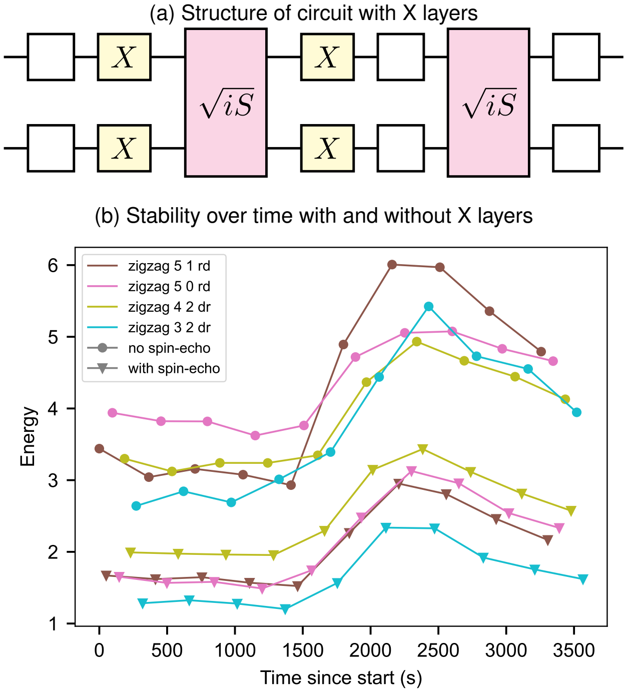

Supplementary Figure 1. (a) Spin-echo technique for reducing errors. (b) The graph shows the energy obtained from measuring at the lowest-energy parameters achievable in VQE depth 1 for a 1×8 instance at half-filling. Lines with triangle markers are with spin-echo, lines with circle markers are without. Exact energy is -3.478 so lower energies are better. 4 sets of qubits were tested in zigzag configurations; labels indicate starting positions and direction ('rd': right then down; 'dr': down then right)

.

2. Symmetries of the Fermi-Hubbard model

The Fermi-Hubbard model has a number of symmetries which can be used to mitigate errors: time-reversal symmetry, particle number conservation per spin sector, particle-hole symmetry, and reflection symmetry of the lattice.

Time-reversal symmetry of the terms in the Fermi-Hubbard model implies that the VQE energy is unchanged if all parameters are negated[45]. This is because the initial state $|\psi_0\rangle$, the ground state of the noninteracting Fermi-Hubbard model, is real in the computational basis, and each of the interaction terms H_k in the Fermi-Hubbard Hamiltonian is symmetric. Hence

$$e^{-i\theta_k H_k} = \left(e^{i\theta_k H_k}\right)^\dagger = \left(e^{i\theta_k H_k}\right)^*,$$

where $*$ denotes complex conjugation in the computational basis. The final state of the algorithm for parameters $\{-\theta_k\}$ is then

$$|\psi_f\rangle = \prod_k e^{-i\theta_k H_k} |\psi_0\rangle = \prod_k (e^{i\theta_k H_k})^* |\psi_0\rangle = \left(\prod_k e^{i\theta_k H_k} |\psi_0\rangle\right)^* .$$

The energy of this state with respect to the overall Fermi-Hubbard Hamiltonian H is then equal to that achieved by the final state with parameters $\{\theta_k\}$, because

$$\langle\psi_f|H|\psi_f\rangle = \langle\psi_f|^* H|\psi_f\rangle^* .$$

We can take advantage of this symmetry by evaluating the energy for a given set of VQE parameters $\boldsymbol{\theta}$ as the average of the energies at $\boldsymbol{\theta}$ and $-\boldsymbol{\theta}$. The intent is that this will mitigate the effect of systematic coherent errors such as over-rotations.

Particle number conservation per spin sector in the Fermi-Hubbard model follows from invariance of the Fermi-Hubbard Hamiltonian under the $U(1) \times U(1)$ symmetry $a^\dagger_{i,\sigma} \mapsto e^{i\alpha_\sigma} a^\dagger_{i,\sigma}$ (i.e. a pair of $U(1)$ transformations, each acting on spin-up and spin-down modes independently). As a consequence, the overall Fermi-Hubbard Hamiltonian preserves the occupation number (Hamming weight) within spin-up and spin-down sectors.

Further, occupation number preservation within each spin sector holds for all operations in the Hamiltonian variational ansatz. As the quantum algorithm starts with a state with a known occupation number in each spin sector, this enables us to reject any runs with an incorrect final occupation number. In particular, this detects many errors that occur due to incorrect qubit readout, which can be a significant source of error in superconducting qubit systems (for example, realistic estimates could be a 1% probability of a 0 being incorrectly read as a 1, and a 5% probability of a 1 being incorrectly read as a 0). We found that in our 16-qubit experiments, between 7% and 29% of runs were retained due to having correct occupation numbers (see Supplementary Table II).

It is interesting to note that we expect that checking the occupation number within each spin sector should be sufficient to detect the vast majority of readout errors, without the need for additional readout error mitigation techniques[46–49]. A rough upper bound on the probability that there is an undetected readout error can be found by multiplying the probability that there is a pair of errors $0 \mapsto 1$ and $1 \mapsto 0$ by the number of pairs of qubits within each spin sector, and then by 2 for the number of spin types. Assuming independent readout errors, the probability of such a pair of errors can be roughly upper-bounded by 10^{-3}, and in a 16-qubit experiment we have 8 qubits in each spin sector, giving $\binom{8}{2} = 28$ possible pairs of readout errors in each spin sector, and hence an overall upper bound of less than 6% on the probability that there is a undetected readout error.

Lattice	Min probability	Max probability
1×4	0.32	0.66
1×8	0.12	0.29
2×4	0.077	0.25

Supplementary Table II. Probabilities of successfully postselecting on occupation number for the different lattices considered in our experiments.

Particle-hole symmetry relates low to high fillings in the Fermi-Hubbard model. On a bipartite lattice we define two sublattices A and B, such that each neighbour of a site in A belongs to B and vice versa. The particle-hole transformation $\mathcal{P}$ acts as $\mathcal{P} a_{j\sigma} \mathcal{P}^\dagger = (-1)^{b_j} a^\dagger_{j\sigma}$, where $b_j = 0$ if $j \in A$ and $b_j = 1$ if $j \in B$. Under this transformation the Hamiltonian (1) in the main text becomes

$$\mathcal{P} H \mathcal{P}^\dagger = H + U(L - N_{\text{occ}}) \tag{3}$$

where $N_{\text{occ}} = N_\uparrow + N_\downarrow$ is the number of electrons in the system and L is the size of the system. The density operator $n_{i\sigma} = a^\dagger_{i\sigma} a_{i\sigma}$ transforms as $\mathcal{P} n_{i\sigma} \mathcal{P}^\dagger = a_{i\sigma} a^\dagger_{i\sigma} = 1 - n_{i\sigma}$. For a unique ground state $|GS_{(N_\uparrow, N_\downarrow)}\rangle$ with N_σ electrons of spin σ, $\mathcal{P}|GS_{(N_\uparrow,N_\downarrow)}\rangle = |GS_{(L-N_\uparrow, L-N_\downarrow)}\rangle$ and the density correlations satisfy

$$\begin{aligned}\langle n_{i\sigma} n_{j\sigma'}\rangle_{N_\uparrow,N_\downarrow} &= \langle n_{i\sigma} n_{j\sigma'}\rangle_{L-N_\uparrow,L-N_\downarrow} \\ &- \langle n_{i\sigma}\rangle_{L-N_\uparrow,L-N_\downarrow} - \langle n_{j\sigma'}\rangle_{L-N_\uparrow,L-N_\downarrow} + 1,\end{aligned} \tag{4}$$

where we defined $\langle GS_{(N_\uparrow,N_\downarrow)}|\mathcal{O}|GS_{(N_\uparrow,N_\downarrow)}\rangle =: \langle \mathcal{O}\rangle_{N_\uparrow,N_\downarrow}$. The spin correlations can be obtained from (4).

Importantly, all the terms in the Hamiltonian Variational ansatz are also essentially invariant under this symmetry. With the "snake" Jordan-Wigner ordering shown in Fig. 1 of the main text the particle-hole transformation becomes simply $\mathcal{P} = X^{\otimes N}$, i.e. an X gate acting on each of the $N = 2L$ qubits. Hopping terms in the ansatz commute with $X^{\otimes 2}$, while onsite terms (CPHASE gates) commute up to a Z rotation on both qubits. As the same parameter is used for all such rotations within one layer, this becomes an unobservable global phase within each occupation number subspace. Thus $X^{\otimes N}$ effectively commutes with the entire variational circuit C, implying that for any observable $\mathcal{O}$,

$$\langle\psi|X^{\otimes N} C^\dagger \mathcal{O} C X^{\otimes N}|\psi\rangle = \langle\psi|C^\dagger X^{\otimes N} \mathcal{O} X^{\otimes N} C|\psi\rangle , \tag{5}$$

and hence that we can interpret any observable on a VQE state $|\psi\rangle$ in terms of a related observable on the particle-hole transformed state $X^{\otimes N}|\psi\rangle$.

Particle-hole symmetry can thus be used for error mitigation, by producing an estimate of an observable for the VQE ground state at occupation number N_{occ} as an average of the experimentally obtained value at N_{occ} and the value at $L - N_{\text{occ}}$ (suitably transformed). This effectively replaces the worst-case and best-case errors of this pair with their average.

Reflection symmetry. The site-dependent observables we measure (charge and spin, and the corresponding correlations) are symmetric about reflections of the lattice in the x and y directions. We can obtain a further reduction in worst-case error by averaging these quantities over reflections of the lattice (2 points in the case of a 1D lattice, and 4 points in the case of a 2D lattice).

3. Training with fermionic linear optics

Training with fermionic linear optics (TFLO) is a method proposed by two of us[50] to mitigate errors in quantum algorithms for simulating fermionic systems, which fits into an overall framework initially introduced by Czarnik et al.[51]. The idea is based on producing a set of pairs of noisy and exact energies, which are then used as training data to infer a map from the noisy energy evaluation for the final state produced by VQE to an approximation of the exact energy.

To implement this concept requires a family of quantum circuits which well-represent the error behaviour of the quantum circuit which we actually wish to evaluate. In Ref. 51, the family of circuits used was Clifford circuits, which can be simulated efficiently classically via the Gottesman-Knill theorem. Here, we use fermionic linear optics (FLO) circuits, which can also be simulated efficiently classically[52]. This family of circuits is tailor-made for mitigating errors in VQE for the Fermi-Hubbard model. An FLO circuit starts with a computational basis state, which corresponds to the state produced by applying some creation operators to the vacuum, and contains operations corresponding to time-evolution by quadratic Hamiltonians, via unitary operators of the form $U = e^{iH}$, where $H = \sum_{j,k} h_{jk} a_j^\dagger a_k$.

In the case of VQE with the Hamiltonian variational ansatz[53], all operations in the circuit are either time-evolution by terms in the Fermi-Hubbard Hamiltonian, or preparation of the initial ground state of the non-interacting Fermi-Hubbard model via Givens rotations (which are FLO). Thus almost all operations in the circuit are FLO, except for time-evolution by the onsite terms. Therefore, any VQE circuit where the onsite parameters are set to 0 is an FLO circuit and can be simulated efficiently to benchmark the behaviour of errors in the circuit.

The TFLO method has been successfully applied to VQE for a 2×3 instance of the Fermi-Hubbard model in classical emulation (with a simple depolarising noise model), and to a 1×2 instance on real quantum hardware[50], reducing errors by a factor of 10–30 or more. However, as with other error mitigation techniques, it is unclear in advance how well TFLO will perform in a given experiment, especially for larger instance sizes.

Here we make several optimisations to fine-tune the performance of TFLO for larger system sizes and enable us to achieve high-quality results. As with previous work[50], we use an algorithm based on linear regression to infer the map from noisy to real energies. As justification for this, if the intended quantum state produced by the experiment with variational parameters $\boldsymbol{\theta}$ is $\psi(\boldsymbol{\theta})$ and the noise process that occurs in the quantum computer is a quantum channel that maps

$$\psi(\boldsymbol{\theta}) \mapsto \widetilde{\psi(\boldsymbol{\theta})} = (1-p)\psi(\boldsymbol{\theta}) + p\rho \tag{6}$$

for some fixed quantum state ρ, then the noisy energy is a corresponding linear transformation of the exact energy $\operatorname{tr} H\widetilde{\psi(\boldsymbol{\theta})}$. Some natural error processes occurring in quantum hardware are indeed of this form (such as the depolarising channel applied to the output of the experiment); however, many other errors are not. Nevertheless, in practice we observe an approximately linear relationship between exact and noisy energies (Supplementary Figure 2).

However, one can see that not all points fit perfectly along a line, and we expect systematic and transient errors to occur that lead to certain data points being low-quality. We use the Theil-Sen estimator[54,55] to reduce the effect of these outliers, which is a linear regression algorithm based on taking the median of the slopes between pairs of points.

Next, to further improve the tolerance of this method to noise, we look for a fairly large number (here we choose 16) of tuples of parameters whose corresponding energies are well spread-out, to minimise the effect on the linear fit of systematic or transient errors in computing each individual energy. For the case of 1 VQE layer, FLO circuits have two nonzero VQE parameters for $1 \times L_y$, and three nonzero parameters for $2 \times L_y$. We find parameters whose corresponding exact energies are spread out by searching over energies obtained for a uniformly spaced grid of size 16 for each parameter. The cost of this method grows exponentially with the number of layers, so for 2 or more layers, we instead search over 256 random parameter choices. In all cases, we use efficient classical simulation software previously developed for VQE for the Fermi-Hubbard model[19] to compute these energies. This code uses an exponential-time simulation approach which does not use the efficient algorithm for simulating FLO circuits[52]; however, for the problem sizes considered, it is sufficiently efficient. As larger problem sizes are considered, it will become essential to use a theoretically efficient classical simulation algorithm.

A final important improvement that we make to the previously developed TFLO algorithm is designed to correct for coherent errors. In principle, TFLO with linear regression can correct for decoherence of the form of (6) with very high accuracy. However, an important class of errors which are not corrected in this way are coherent errors that depend on the choice of parameters (for example, over-rotations). To correct for errors of this form, after performing TFLO as previously discussed, we implement a final step where we subtract off the residual error at the FLO point which is closest to the parameters at which we wish to evaluate the energy – the point obtained by setting the onsite parameter(s) to zero. The

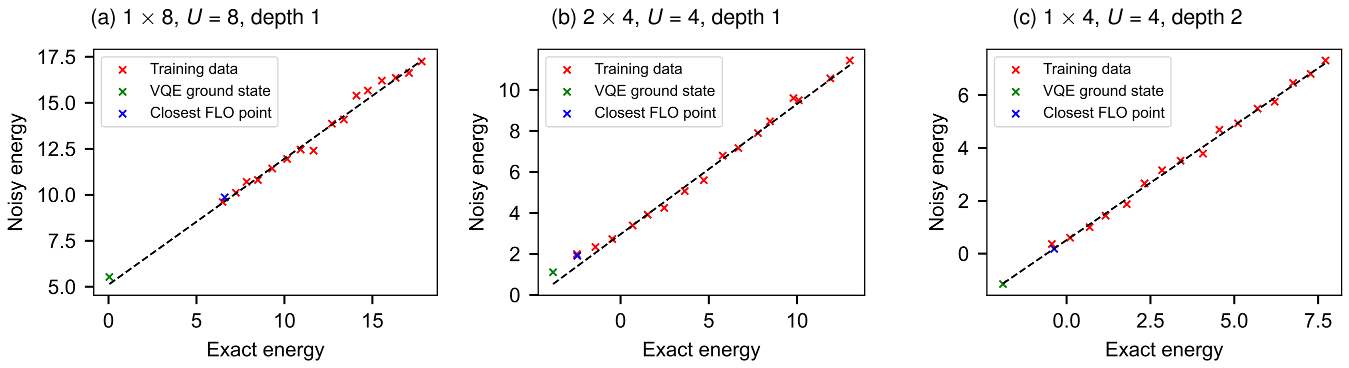

Supplementary Figure 2. Representative examples showing a near-linear relationship between noisy and exact VQE energies for (a) 1×8 (with depth 1), (b) 2×4 (with depth 1), and (c) 1×4 (with depth 2) instances of the Fermi-Hubbard model. Here, $U = 8$ in panel (a) and $U = 4$ in panels (b)–(c). All results are at half-filling, corresponding to some of the most complex circuits we execute. The straight line demonstrates the fit found using the Theil-Sen algorithm we used. Residual error is reduced by subtracting the remaining error in the closest FLO point to the VQE parameters (shown in green). Error bars are too small to be visible.

intuition behind this step is that, as almost all gates in this FLO circuit are the same as the real circuit we wish to execute, we expect the error behaviour to be very similar.

Other observables. As well as the energy, TFLO can be applied to any other observable, as long as the exact and noisy expectations are available for the FLO points. Except for the energy, all observables considered by us are diagonal in the computational basis, which means their noisy expectation values can be approximately computed from the same samples that were used to compute the expectation value of the onsite term in the Hamiltonian. For any diagonal observable, the corresponding exact expectation value can be approximately computed from samples in the computational basis at the FLO point, which can be generated efficiently classically[52]; the observables considered here could also be computed exactly. (In our experiments, for ease of implementation we instead used samples generated by simulating the circuit directly, which is sufficiently efficient for the circuit sizes we consider.)

Hence TFLO can be applied to all other observables considered in this paper as well, without the need for additional quantum resources. However, a caveat applies: The evaluation points for TFLO were chosen such that the energies are spread out to facilitate a good linear fit. This does not necessarily imply that the expectation values of other observables are also spread out.

In fact, at half-filling the ground state expectation value of all single-site density $n_{i\sigma}$ operators is uniformly $\frac{1}{2}$ due to the particle-hole symmetry. And for states with uniform density, the density is invariant under FLO circuits (we will prove this shortly). Hence the inferred linear transformation from noisy densities to exact densities will always be the constant $\frac{1}{2}$-function. Note however, that in this special case of half-filling the particle-hole symmetry holds regardless of whether $U = 0$ and hence predicting $\langle n_{i\sigma}\rangle = \frac{1}{2}$ is actually correct. For other observables (e.g. charge densities) the expectation value of the observable to mitigate is invariant under FLO circuits, but does change when applying non-FLO gates. In such cases TFLO would fail altogether and would always predict the value of the FLO simulations. To avoid these issues, we performed the following checks. First, if the exact observable values found by classical simulation were all close (within 0.05), we only applied the coherent error correction step, and not a linear fit (which would be meaningless in this case). Otherwise, we performed a linear fit using the Theil-Sen algorithm, and checked whether the linear fit was a good explanation of the data, as measured by the coefficient of determination (R^2) being larger than 0.7. If this check failed, we assume that there is no simple relationship between the noisy and exact values and simply return the noisy value of the observable.

The fact that the charge and spin densities of states with constant density are invariant under FLO circuits can be shown as follows. First note that any (number-preserving) FLO unitary can be written as the product of Givens rotations, hence it suffices to show the statement for Givens rotations. Without loss of generality, consider a Givens rotation applied to the first two qubits, write the state as

$$|\Psi\rangle = |00\rangle\,|\psi_{00}\rangle + |01\rangle\,|\psi_{01}\rangle + |10\rangle\,|\psi_{10}\rangle + |11\rangle\,|\psi_{11}\rangle \quad (7)$$

and note that $\langle n_1\rangle_\Psi = \langle n_2\rangle_\Psi$ implies $\langle\psi_{01}|\psi_{01}\rangle + \langle\psi_{11}|\psi_{11}\rangle = \langle\psi_{10}|\psi_{10}\rangle + \langle\psi_{11}|\psi_{11}\rangle \Leftrightarrow \langle\psi_{01}|\psi_{01}\rangle = \langle\psi_{10}|\psi_{10}\rangle$. Applying a Givens rotation with angle θ to the first two qubits then yields

$$\begin{aligned} |\Psi'\rangle &= G_{12}(\theta)\,|\Psi\rangle \\ &= |00\rangle\,|\psi_{00}\rangle + [\cos(\theta)\,|01\rangle + \sin(\theta)\,|10\rangle]\,|\psi_{01}\rangle \\ &\quad + [-\sin(\theta)\,|01\rangle + \cos(\theta)\,|10\rangle]\,|\psi_{10}\rangle + |11\rangle\,|\psi_{11}\rangle \end{aligned} \quad (8)$$

and computing the density on the first qubit then yields

$$\langle n_1 \rangle_{\Psi'} = \cos(\theta)^2 \langle \psi_{01} | \psi_{01} \rangle + \sin(\theta)^2 \langle \psi_{10} | \psi_{10} \rangle + \langle \psi_{11} | \psi_{11} \rangle = \langle n_1 \rangle_{\Psi} \quad (9)$$

where we used for the second equality that $\langle \psi_{01} | \psi_{01} \rangle = \langle \psi_{10} | \psi_{10} \rangle$. Similarly $\langle n_2 \rangle_{\Psi'} = \langle n_2 \rangle_{\Psi}$. For all other sites j the density is invariant because $[n_j, G_{12}(\theta)] = 0$. Hence $\langle n_i \rangle$ is invariant under FLO unitaries.

4. Sampling overheads and scalability

In this section we discuss the overheads associated with our error-mitigation techniques, and their scalability. Among the low-level techniques, the only one with an overhead is selecting the best of 3 runs. The use of time-reversal symmetry has a running time overhead of a factor of 2, because each set of VQE parameters is mapped to two sets. Similarly, taking advantage of particle-hole symmetry involves doubling the number of runs. The use of reflection symmetry has no additional cost.

For particle number conservation, experimental sampling overheads are listed in Supplementary Table II. One can use known characterisations of the probability of readout errors to estimate the sampling overhead for larger system sizes. Google's 53-qubit Weber processor has estimated $0 \mapsto 1$ readout error rate 2% (when measuring in parallel), and $1 \mapsto 0$ readout error rate 7%. Assuming that these error rates extend to larger systems, at half-filling a 64-qubit experiment (beyond the bounds of classical exact simulability) would have a 17% expected probability of retaining each run; a 128-qubit experiment would have a 7% probability of retention. These are not unreasonable overheads in runtime.

The overhead associated with TFLO corresponds to the evaluation of FLO circuits for a constant number of fixed parameter settings (here we used 16), as well as for a choice of parameter settings associated with the point at which the energy is evaluated.

For large Fermi-Hubbard lattice sizes, a more significant source of error than qubit readout will be errors associated with imperfect 2-qubit gates. Our experiments used up to 176 2-qubit gates on a quantum processor with 2-qubit gate fidelities ~ 0.995. Converting this to a roughly estimated quantum circuit fidelity by simply exponentiating the gate fidelity by the number of gates gives an estimated circuit fidelity ~ 0.41, which was sufficient to achieve qualitatively accurate results.

Implementing one layer of the Hamiltonian variational ansatz for a $1 \times L_y$ lattice (which is classically solvable) using the quantum circuit family proposed in this work requires $2 \cdot (2L_y + 2(L_y - 1)) = 4(2L_y - 1)$ $\sqrt{\text{iSWAP}}$ gates in a quantum circuit of depth 10. At most L_y^2 2-qubit gates are also required to prepare the initial state, using a quantum circuit of depth $2(L_y - 1)$. Finally, at most $2L_y$ 2-qubit gates are used for the final energy measurement, in depth 2. The total 2-qubit gate complexity for a 1-layer VQE circuit is then at most $L_y^2 + 10L_y - 4$ in depth $2L_y + 10$. This inevitably leads to circuit fidelities dropping exponentially with the instance size; however, even for quite large instances, the overall circuit fidelity may still be reasonably high. Addressing a 1×25 Fermi-Hubbard instance, for example, would use 871 gates, and with a gate fidelity of 0.995, an estimated circuit fidelity of ~ 0.01 would be achieved.

Even a fidelity this small could be sufficient to achieve qualitatively accurate results when enhanced using TFLO and other error-mitigation techniques, although experimentation would be required to determine the level of accuracy that could be achieved, and the overheads incurred. One might anticipate overheads to increase inversely proportionally to the circuit fidelity. A larger device allowing the ability to run multiple copies of the VQE circuit would enable the use of additional error-mitigation techniques such as virtual distillation/exponential error suppression[56,57], at the cost of an overhead in quantum circuit depth and number of measurements. These techniques could be combined with the error-mitigation techniques used here.

Addressing $L_x \times L_y$ instances for $L_x > 1$, or using larger numbers of variational ansatz layers, will require more complex circuits and hence higher-performance hardware. For an $L_x \times L_y$ lattice, the gate complexity scaling of the algorithm proposed in Ref. 19 is $O(L_x L_y (L_x L_y + k L_x))$ for a circuit with k variational layers. On the other hand, the circuit depth of this algorithm is substantially smaller, by a factor of $O(L_x L_y)$, which may significantly improve the accuracy of estimating physically relevant observables in practice.

Supplementary Note 5 – Classical comparators

In this section we give more details about how the classical comparator quantities in Figures 4, 5, and 6 in the main text and elsewhere were calculated.

Simulated and ground state quantities. The "simulated" line in Fig. 4 in the main text refers to the energy produced using an exact classical simulation of the VQE algorithm, using a software package targeted at efficient simulation of the Fermi-Hubbard model[19]. The optimisation algorithm used was BFGS, which was previously found to be effective[19], but can get trapped in local minima; results were therefore checked using the Cobyla algorithm. Ground state quantities such as energies, charges, spins, and correlations were computed by exact diagonalisation.

Slater determinant. A natural classical comparator against the performance of the variational ansatz of quantum states that we use is the family of Slater determinant (Hartree-Fock) states, which can be seen as product states that obey appropriate fermionic antisymmetry.

Given a Slater determinant, its energy with respect to the Fermi-Hubbard Hamiltonian can be computed ef-

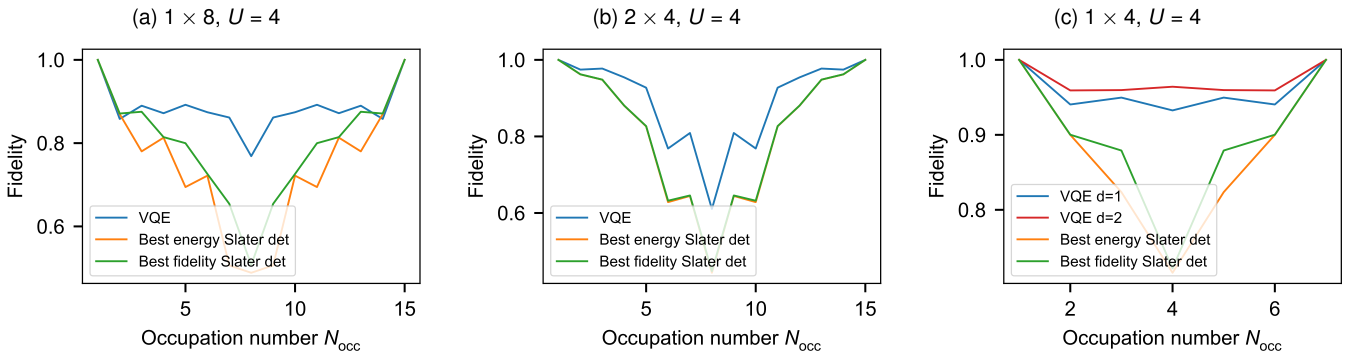

Supplementary Figure 3. Fidelity $|\langle\widetilde{\psi_G}|\psi_G\rangle|^2$ with the ground state $|\psi_G\rangle$ achieved by the VQE estimate $|\widetilde{\psi_G}\rangle$ (in theory), compared with the best achievable fidelity by a Slater determinant, and the fidelity achieved by the Slater determinant with the lowest energy for the (a) 1×8, (b) 2×4, and (c) 1×4 Fermi-Hubbard instances. VQE results in (a)–(b) use one variational layer, while in (c) results for both one (red) and two (blue) layers are shown. In all panels, $U=4$.

ficiently either using a general technique for simulating fermionic linear optics circuits[52], or more simply via the Slater-Condon rules from quantum chemistry. One particular Slater determinant that can be used as a trial state is the ground state of the $U=0$ (noninteracting) Fermi-Hubbard model. However, it is possible to achieve an energy closer to the true ground energy, for example by the well-known mean-field approximation to the Fermi-Hubbard model[58].

The iterative mean-field approach is not guaranteed to converge, or to find a Slater determinant that minimises the energy. To measure the ability of a "best possible" Slater determinant to compete with the VQE solution, we therefore used a different approach, where we optimised (classically) over the space of Slater determinants, with the goal of minimising the energy with respect to the Fermi-Hubbard Hamiltonian. To parametrise this space, we used the entries of an $L\times L$ Hermitian matrix h for a system with L sites, corresponding to a Hamiltonian $H_{SD}=\sum_{i,j} h_{ij}a_i^\dagger a_j$, with the same matrix h being used for spin-up and spin-down, to ensure that spin-flip symmetry was obeyed. Then the Slater determinant with occupation number k corresponding to this matrix, the ground state of H_{SD}, is found by taking the k eigenvectors $e_i=(\alpha_{i1},\dots,\alpha_{iL})$ of h with lowest eigenvalues and forming the product of single-particle operators $\alpha_{i1}a_1^\dagger+\cdots+\alpha_{iL}a_L^\dagger$. In the case of even occupation numbers, we used the same occupation number for spin-up and spin-down; for odd occupation numbers, we had one more spin-up electron (matching the VQE experiments).

We used the Slater-Condon rules to compute the overall Fermi-Hubbard energy corresponding to h, as a subroutine within the classical BFGS optimiser. This allowed us to minimise this energy over h, for several randomly perturbed starting conditions. We found that this approach reliably converged to the apparent global minimum.

Fidelities. A more stringent condition on preparation of the ground state than the energy being low is that the fidelity with the ground state is high. The fidelity is not accessible experimentally, but the best achievable fidelity of the VQE approximation to the ground state can be computed in classical emulation and compared against the best possible fidelity achievable by a Slater determinant. These results are shown in Supplementary Figure 3; in almost all cases the VQE approximation outperforms the best possible Slater determinant.

Supplementary Note 6 – Formula for variance of observables taking postselection into account

In this section we derive the expression stated in Ref. 59 for the variance of the sample mean when the number of samples is random. Assume that $X_1,\cdots,X_N$ are N i.i.d random variables with mean μ and variance σ^2 and $Y_1,\cdots,Y_N$ are N i.i.d. Bernoulli variables with $p(Y_i=1)=p$ for all i. The X_i play the role of our samples and Y_i indicates if we keep the i-th sample after postselection or not. The estimator for the mean μ is then

$$Z=\frac{\sum_i Y_i X_i}{\sum_j Y_j}. \tag{10}$$

It is straightforward to verify that this estimator is unbiased:

$$\mathbb{E}[Z]=\mathbb{E}\left[\frac{\sum_i Y_i X_i}{\sum_i Y_i}\right]=\sum_i \mathbb{E}[X_i]\mathbb{E}\left[\frac{Y_i}{\sum_j Y_j}\right]=\mu. \tag{11}$$

To calculate its variance, it is useful to condition on the number of successful samples $|Y|=\sum_i Y_i$:

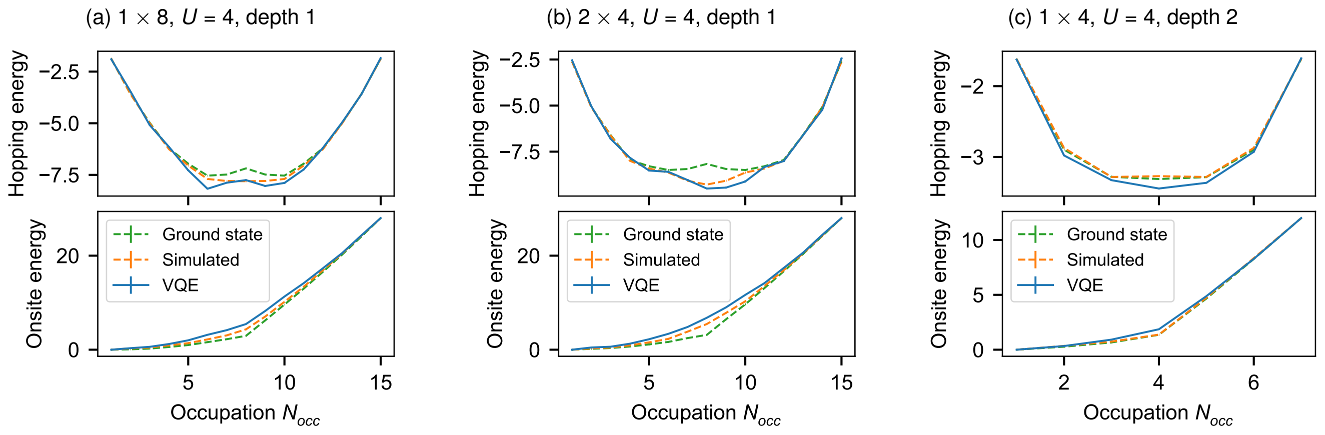

Supplementary Figure 4. Comparison of the hopping (kinetic) and onsite (potential) energies in the experimental VQE ground state (blue, solid lines), the perfect VQE ground state found in classical simulations (yellow, dashed lines), and the true ground state for the three lattices studied by us (green, dashed lines) for (a) 1×8, (b) 2×4, and (c) 1×4 Fermi-Hubbard instance. Results shown in (a)–(b) have been obtained with a single variational layer, while in (c) two layers have been used. In all panels, $U = 4$.

$$\begin{aligned}\mathbb{E}[Z^2] &= \sum_y \frac{\Pr[Y=y]}{|y|^2}\mathbb{E}_X\Big[\Big(\sum_{i\,:\,y_i=1} X_i\Big)^2\Big] \\ &= \sum_y \frac{\Pr[Y=y]}{|y|^2}\mathbb{E}_X\left[\sum_{i\,:\,y_i=1} X_i^2 + \sum_{i\neq j\,:\,y_i=y_j=1} X_iX_j\right] \\ &= \sum_y \frac{\Pr[Y=y]}{|y|^2}\left(|y|\mathbb{E}[X_1^2] + |y|(|y|-1)\mathbb{E}[X_1]^2\right) \\ &= \mathbb{E}[X_1]^2 + \sum_y \frac{\Pr[Y=y]}{|y|}(\mathbb{E}[X_1^2]-\mathbb{E}[X_1]^2) \\ &= \mu^2 + \mathbb{E}[1/|Y|]\sigma^2,\end{aligned} \tag{12}$$

so

$$\mathrm{Var}(Z) = \mathbb{E}[Z^2] - \mathbb{E}[Z]^2 = \sigma^2\mathbb{E}_Y[1/|Y|]. \tag{13}$$

To calculate the remaining expectation value, note that in all our cases $|Y|$ is large (around 20,000) and hence well concentrated. A straightforward Taylor expansion of $f(|Y|) = \frac{1}{|Y|}$ around its expectation value Np yields then

$$\frac{1}{|Y|} = \frac{1}{Np} - \frac{|Y|-Np}{(Np)^2} + \frac{(|Y|-Np)^2}{(Np)^3} + O\left((Np)^{-4}\right) \tag{14}$$

and taking expectation values on both sides gives

$$\mathbb{E}_Y\left[\frac{1}{|Y|}\right] = \frac{1}{Np} - 0 + \frac{Np(1-p)}{(Np)^3} \tag{15}$$

which finally makes the variance

$$\mathrm{Var}(Z) = \frac{\sigma^2}{Np}\left(1 + \frac{1-p}{Np}\right) + O\left((Np)^{-3}\right). \tag{16}$$

Several more complicated and accurate asymptotic expansions are given in Ref. 59 and references therein. The above expression is sufficient for our needs (indeed, the $O(1/(pN)^2)$ correction is already almost unnoticeable).

Supplementary Note 7 – Additional experimental results

1. Hopping and onsite energies

In Supplementary Figure 4 we repeat the analysis done for Fig. 4 of the main text, but this time splitting the kinetic (hopping) and interaction (onsite) contributions to the energy. Comparing the "Simulated" with the "Ground state" energies one can see that the VQE state overestimates the onsite energy, but underestimates the hopping energy. This leads to some cancellation of errors, which helps to achieve the very small overall energy errors reported in the main text. One can interpret this as the VQE ground state lying "between" the true ground state and the initial noninteracting ground state.

However, error cancellation does not fully explain the accuracy of our results: for example, for the 1x4 lattice at half-filling, the hopping energy error between our experimental results and the ground state is −0.133, the onsite energy error is 0.505, yet the overall energy error is 0.046. This is because our error-mitigation methods act on the energy as a whole, enabling higher-accuracy results than acting on each energy separately. Higher depth VQE circuits would produce more accurate approximations, and would eventually reproduce the exact hopping and onsite energy curves.

Furthermore, in Supplementary Figure 4 the "VQE" hopping energy and the "Simulated" hopping energy are in better agreement than the respective onsite energies.

This can be explained by the TFLO training points having the same onsite energy, making a linear fit impossible. This means TFLO does not work effectively to correct errors in the onsite energy.

2. Additional results for the 2×4 lattice

In this section we collate figures and additional discussion for our results on a 2×4 lattice.

Metal-insulator transition. In Supplementary Figure 5 (a)-(b), we observe the behaviour of the chemical potential for different occupations. We do not see the onset of the MIT, as μ' remains essentially constant. We suspect that this is due to two compounded effects: uncorrected errors affecting the quantum processor, and the low depth of the EHV ansatz used not being able to sufficiently capture correlations in this system, as we discuss below.

Charge density expectation. In large 2D systems, it is expected that at half-filling the charge becomes commensurate with the lattice, spontaneously breaking discrete translation symmetry. With this in mind we study the charge density in the 2×4 system (Supplementary Figure 6(a),(e)). In this small system we find that the charge expectation inherits some of the physics of 1D. A way of seeing this is the following. Consider the non-interacting limit $U = 0$ with zero hopping along the rungs ($t_R = 0$). In this case the energy of the system consists of two degenerate bands each corresponding to a 1×4 system. Increasing the hopping along the rungs to its original $t_R = 1$ value, the two degenerate bands split in energy by t_R. The single particle states in the lower band correspond to the symmetric combinations of electrons from both chains. Increasing the interactions from zero gradually, the system resembles a 1×4 Hubbard chain of fermions delocalised along the rungs. This explains the uniform charge density profile at a quarter filling ($N_{\text{occ}} = 4$), as this corresponds to a completely filled lower band of "rung" electrons. This delocalisation also decreases the effect of the onsite interaction, as can be seen by comparing a true 1D system with the effective one made of "rung" electrons using standard perturbation theory around the non-interacting regime.

Spin density. The spin profiles for odd occupations for the VQE ansatz (Supplementary Figure 6(c)–(d)) and for the true ground state (Supplementary Figure 6(g)-(h)) are similar, and close to the non-interacting pattern (Supplementary Figure 6(f)). This supports the interpretation that VQE is able to capture non-correlated behaviour at depth 1 in this system, to reproduce the physics of a weakly interacting system, but fails to account for correlations, which in this system are "screened" by the delocalisation of particles across the rungs. Based on this, we expect that to observe truly correlated behaviour in this system (e.g. a peak in μ' around half-filling, Supplementary Figure 6), we need a deeper VQE circuit to represent the ground state observables with higher accuracy.

Supplementary Note 8 – Further details about BayesMGD

Here, we give an explicit formulas for $\boldsymbol{\beta}_m$ and $\boldsymbol{\Sigma}_m$ in Eq. (5) of the main text together with their derivation. We also present pseudo-code for the BayesMGD algorithm and show how it is related to Kalman filters.

1. The Bayesian update step

We make three main assumptions in our design of the BayesMGD algorithm:

1. For all $\boldsymbol{\theta}'$ near $\boldsymbol{\theta}_{m-1}$ the probability of observing some value y' is given by $p(y' \,|\, \boldsymbol{\theta}', \boldsymbol{\beta}) = \mathcal{N}(f_s(\boldsymbol{\theta}'; \boldsymbol{\beta}))$ where the surrogate model can be written as $f_s(\boldsymbol{\theta}'; \boldsymbol{\beta}) = \sum_j^{n_m} \beta_j \phi_j(\boldsymbol{\theta})$ with n_m *model functions* $\phi_j(\boldsymbol{\theta})$ that can be read off Eq. (4) of the main text.

2. In the m-th iteration, before observing new data our belief about the model parameters is a multivariate Gaussian $p_{m|m-1}(\boldsymbol{\beta}) = \mathcal{N}(\boldsymbol{\beta}_{m|m-1}, \boldsymbol{\Sigma}_{m|m-1})$.

3. When making a gradient descent step, we lose certainty about the model parameters $\boldsymbol{\beta}$ proportional to the step width s, but our belief does not change. That is, making the gradient descent step sends $\boldsymbol{\Sigma}_{m+1|m} = \boldsymbol{\Sigma}_{m|m} + \frac{s^2}{l^2}\mathbb{1}$ and $\boldsymbol{\beta}_{m+1|m} = \boldsymbol{\beta}_{m|m}$.

Assumptions 1 and 2 imply that after observing new data $\{y_i, \sigma_i\}$ at points $\{\boldsymbol{\theta}^{(i)}\}$ we can use Bayes' theorem (compare Eq. (5) of the main text) to compute the posterior of $\boldsymbol{\beta}$,

$$\begin{aligned} p_{m|m}(\boldsymbol{\beta}) \propto & P(\{y_i, \sigma_i\} \,|\, \{\boldsymbol{\theta}^{(i)}\}, \boldsymbol{\beta}) \, p_{m|m-1}(\boldsymbol{\beta}) \\ = & \prod_{i=1}^{n_p} \mathcal{N}(y_i; f_s(\boldsymbol{\theta}^{(i)}; \boldsymbol{\beta}), \sigma_i) \\ & \times \mathcal{N}(\boldsymbol{\beta}; \boldsymbol{\beta}_{m|m-1}, \boldsymbol{\Sigma}_{m|m-1}) \\ = & \mathcal{N}(\boldsymbol{\beta}; \boldsymbol{\beta}_{m|m}, \boldsymbol{\Sigma}_{m|m}), \end{aligned} \tag{17}$$

Defining the *design matrix*

$$\mathbf{X} = \begin{pmatrix} \phi_1(\boldsymbol{\theta}^{(1)}) & \cdots & \phi_{n_m}(\boldsymbol{\theta}^{(1)}) \\ \vdots & & \vdots \\ \phi_1(\boldsymbol{\theta}^{(n_p)}) & \cdots & \phi_{n_m}(\boldsymbol{\theta}^{(n_p)}) \end{pmatrix}, \tag{18}$$

the *measurement noise matrix*

$$\boldsymbol{\Sigma} = \begin{pmatrix} \sigma_1^2 & & \\ & \ddots & \\ & & \sigma_{n_p}^2 \end{pmatrix} \tag{19}$$

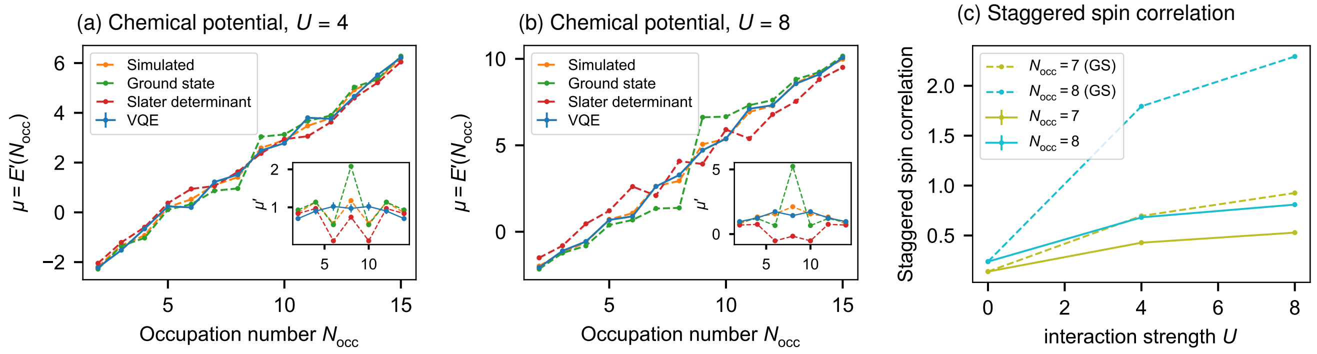

Supplementary Figure 5. Additional results for 2×4. (a)–(b): Chemical potentials $\mu(N_{\mathrm{occ}}) = E(N_{\mathrm{occ}}) - E(N_{\mathrm{occ}} - 1)$. Labels are as in Fig. 4 in the main text. Inset shows the derivative $\mu'(N_{\mathrm{occ}}) = E(N_{\mathrm{occ}} + 1) - 2E(N_{\mathrm{occ}}) + E(N_{\mathrm{occ}} - 1)$ of the chemical potential at even occupations. (c): Total staggered spin correlation $\sum_s (-1)^s \langle S_s^z S_{s+1}^z \rangle$ at and close to half-filling, where the ordering is taken along the Jordan-Wigner snake (Fig. 1(a) in the main text). It compares experimental results from VQE with true values in the ground state (GS). $U = 0$ points are exact classical calculations.

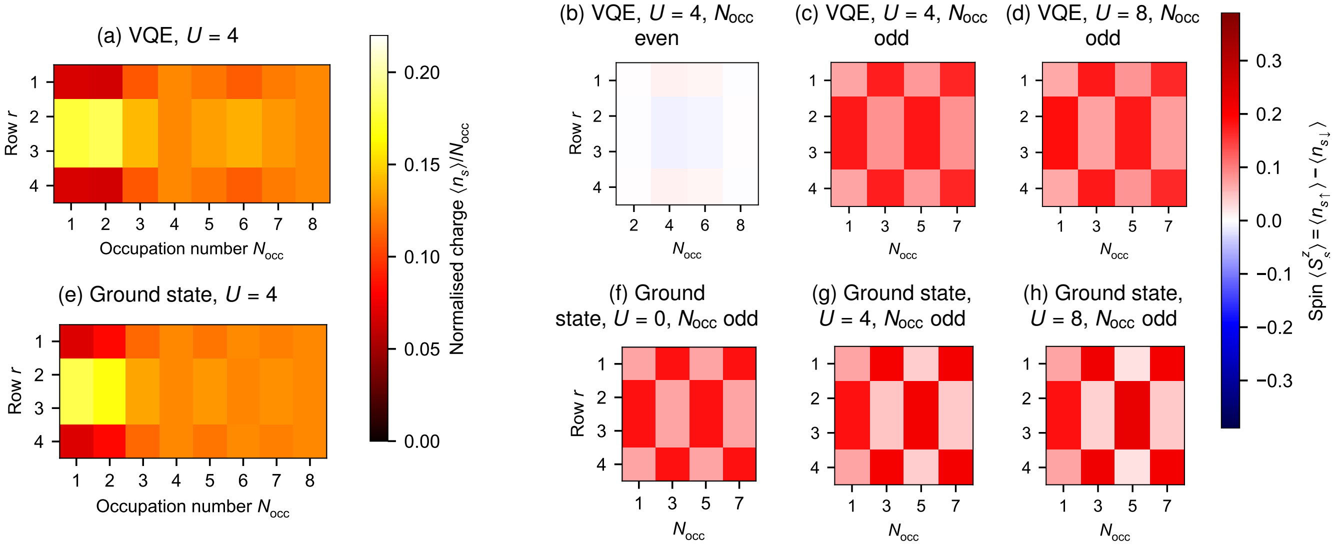

Supplementary Figure 6. (a) Experimental VQE and (e) ground state charge density for a 2×4 lattice. (b)–(d) Experimental VQE and (f)–(h) ground state spin density for a 2×4 lattice. In all panels, the X axis shows the occupation number N_{occ} while the Y axis the row index. By symmetry, charges and spins are equal for each pair of sites in each row, so the plot shows charge/spin for one site in each row. Spin plots split by even/odd occupations. In the ground state, spin is 0 everywhere for N_{occ} even. In the VQE ground state (b), spin is close to 0 everywhere.

and a *measurement outcome vector*

$$\mathbf{y} = (y_1, \cdots, y_{n_p})^\dagger, \tag{20}$$

and plugging these into the expressions for Gaussian distibutions yields

$$\begin{aligned} \log p_{m|m}(\boldsymbol{\beta}) = &-\frac{1}{2}(\mathbf{y} - \mathbf{X}\boldsymbol{\beta})^\dagger \boldsymbol{\Sigma}^{-1}(\mathbf{y} - \mathbf{X}\boldsymbol{\beta}) \\ &- \frac{1}{2}(\boldsymbol{\beta} - \boldsymbol{\beta}_{m|m-1})^\dagger \boldsymbol{\Sigma}_{m|m-1}^{-1}(\boldsymbol{\beta} - \boldsymbol{\beta}_{m|m-1}) \\ &+ \mathrm{const}, \end{aligned} \tag{21}$$

where the normalisation factors were absorbed into "const". The product of two Gaussian distributions is again a Gaussian distribution. So we know that

$$\log p_{m|m}(\boldsymbol{\beta}) = -\frac{1}{2}(\boldsymbol{\beta} - \boldsymbol{\beta}_{m|m})^\dagger \boldsymbol{\Sigma}_{m|m}^{-1}(\boldsymbol{\beta} - \boldsymbol{\beta}_{m|m}) + \mathrm{const} \tag{22}$$

for some $\boldsymbol{\beta}_{m|m}$ and $\boldsymbol{\Sigma}_{m|m}$. Comparing terms in the last two equations then gives

$$\begin{aligned} \boldsymbol{\Sigma}_{m|m}^{-1} &= \mathbf{X}^\dagger \boldsymbol{\Sigma}^{-1}\mathbf{X} + \boldsymbol{\Sigma}_{m|m-1}^{-1} \\ \boldsymbol{\beta}_{m|m} &= \boldsymbol{\Sigma}_{m|m}(\mathbf{X}^\dagger \boldsymbol{\Sigma}^{-1}\mathbf{y} + \boldsymbol{\Sigma}_{m|m-1}^{-1}\boldsymbol{\beta}_{m|m-1}). \end{aligned} \tag{23}$$

Algorithm 1: BayesMGD

Input: function f returning uncertain values y, σ_y,
model functions $\{\phi_j\}_{j=1}^{n_m}$,
initial point $\boldsymbol{\theta}$, stability constant A,
learning rate γ, rate decay exponent α,
sampling radius δ, radius decay exponent ξ,
sample number n_p, maximum evaluations n_{eval},
tolerance ϵ, length scale l,
initial parameters $\boldsymbol{\beta}$, covariance matrix $\boldsymbol{\Sigma}$

$m \leftarrow 0$
while $m < n_{\text{eval}}$ **do**
  $m \leftarrow m + 1$
  $\delta_m \leftarrow \delta / m^{\xi}$ ▷ Set sample radius
  $\gamma_m \leftarrow \gamma/(m + A)^{\alpha}$ ▷ and step width
  $S \leftarrow$ sample n_p points from δ_m-ball around $\boldsymbol{\theta}$
  $L \leftarrow \{\}$
  for $\boldsymbol{\theta}_i \in S$ **do**
    Add $f(\boldsymbol{\theta}_i) = (y_i, \sigma_i)$ to L
  end
  $(\boldsymbol{\beta}, \boldsymbol{\Sigma}) \leftarrow$ BayesUpdate$(\{\phi_j\}, S, L, \boldsymbol{\beta}, \boldsymbol{\Sigma})$
  $\mathbf{g} \leftarrow \nabla_{\boldsymbol{\theta}} f_s(\boldsymbol{\theta}; \boldsymbol{\beta})$
  $\boldsymbol{\theta} \leftarrow \boldsymbol{\theta} - \gamma_m \mathbf{g}$ ▷ Gradient descent step
  $\boldsymbol{\Sigma} \leftarrow \boldsymbol{\Sigma} + \frac{|\gamma_m \mathbf{g}|^2}{l^2} \mathbb{1}$ ▷ Add uncertainty due to step
  if $\gamma_m \cdot |\mathbf{g}| < \epsilon$ **then**
    $(y, \sigma_y) \leftarrow f_s(\theta; \boldsymbol{\beta}, \boldsymbol{\Sigma})$
    return $((y, \sigma_y), \boldsymbol{\theta})$ ▷ Return if step size is small
  end
end
$(y, \sigma_y) \leftarrow f_s(\theta; \boldsymbol{\beta}, \boldsymbol{\Sigma})$
return $((y, \sigma_y), \boldsymbol{\theta})$ ▷ Return if maximum iterations reached

Algorithm 2: BayesUpdate

Input: model functions $\{\phi_j\}_{j=1}^{n_m}$,
evaluation points $\{\boldsymbol{\theta}_i\}_{i=1}^{N}$,
values with noise $\{(y_i, \sigma_i)\}_{i=1}^{N}$,
prior parameters with covariance $\boldsymbol{\beta}_0, \boldsymbol{\Sigma}_0$

Create empty $n_m \times N$ matrix $\mathbf{X}$ ▷ Data preparation
$X_{ji} \leftarrow \phi_j(\boldsymbol{\theta}_i)$
Collect the y_i into $\mathbf{y}$
Collect the σ_i^2 onto diagonal of $\boldsymbol{\Sigma}$
$\boldsymbol{\Sigma}_1^{-1} \leftarrow \mathbf{X}^{\dagger} \boldsymbol{\Sigma}^{-1} \mathbf{X} + \boldsymbol{\Sigma}_0^{-1}$ ▷ Update equations
$\boldsymbol{\beta}_1 \leftarrow \boldsymbol{\Sigma}_1 (\mathbf{X}^{\dagger} \boldsymbol{\Sigma} \mathbf{y} + \boldsymbol{\Sigma}_0^{-1} \boldsymbol{\beta}_0)$
return $(\boldsymbol{\beta}_1, \boldsymbol{\Sigma}_1)$

2. Pseudocode for BayesMGD

In Algorithms 1 and 2 we give pseudocode for the BayesMGD algorithm and the Bayesian update step. Throughout we assume a linear model of the form $f_s(\boldsymbol{\theta}; \boldsymbol{\beta}) = \sum_{j=1}^{n_m} \beta_j \phi_j(\boldsymbol{\theta})$, where n_m is the number of model parameters and the model is linear in the model parameters β_j, but the model functions ϕ_j are not necessarily linear in $\boldsymbol{\theta}$. Compare this with Eq. (4) of the main text,

$$f_s(\boldsymbol{\theta}; \boldsymbol{\beta}) = \beta_0 + \sum_{j=1}^{n_c} \beta_j \theta_j + \sum_{j,k=1,\ j<k}^{n_c} \beta_{jk} \theta_j \theta_k, \tag{24}$$

to identify the model functions $\phi_j(\boldsymbol{\theta})$. For simplicity of notation, we also implicitly lift any function that is passed a variable together with the uncertainty of the variable to a version that returns the function value together with its uncertainty computed using Gaussian error propagation.

3. Relation between BayesMGD and Kalman filters

The Kalman filter is an algorithm that iteratively combines noisy measurement data with prior knowledge and knowledge of the dynamics to estimate the state of a dynamical system[60]. A notable, early application was in the Apollo Guidance Computer[61]. However, as far as we know, Kalman filters have not yet been considered in the context of function optimisation. Instead their main application so far was to estimate the state of physical systems, like air- or spacecraft and robotics. We will now show that our BayesMGD algorithm is mathematically equivalent to the Kalman filter algorithm.

In the language of Kalman filters,

- $\mathbf{x}_{m-1|m-1}$ is the state estimate at time step m given all measurements up to and including time step $m-1$. In our case these are the most likely model parameters $\boldsymbol{\beta}_{m-1|m-1}$;
- $\mathbf{P}_{m-1|m-1}$ is the covariance matrix of that estimate. In our case this is $\boldsymbol{\Sigma}_{m-1|m-1}$.

From these the state at time m is predicted as

$$\begin{aligned} \mathbf{x}_{m|m-1} &= \mathbf{F}_m \mathbf{x}_{m-1|m-1} + \mathbf{B}_m \mathbf{u}_m \\ \mathbf{P}_{m|m-1} &= \mathbf{F}_m \mathbf{P}_{m-1|m-1} \mathbf{F}_m^{\dagger} + \mathbf{Q}_m \end{aligned} \tag{25}$$

where $\mathbf{F}_m$ encodes the dynamics of the system, $\mathbf{B}_m \mathbf{u}_m$ is the result of external control inputs and $\mathbf{Q}_m$ is the process noise. In our case the dynamics are trivial ($\mathbf{F}_m = \mathbb{1}$), there is no external control input $\mathbf{u}_m$ and the process noise is $\mathbf{Q}_m = \frac{s^2}{l^2}$. In the next step, observation data is used to refine the prediction. The observations $\mathbf{z}_m$ are linearly related to the true state $\mathbf{x}_m$ as

$$\mathbf{z}_m = \mathbf{H}_m \mathbf{x}_m + \mathbf{v}_m \tag{26}$$

where $\mathbf{H}_m$ is some (possibly m-dependent) matrix and $\mathbf{v}_m$ the observation noise which is assumed to be zero-mean gaussian white noise $\mathbf{v}_m \sim \mathcal{N}(0, \mathbf{R}_m)$. In our case the observation data are the measurement outcomes $\mathbf{y}$, the measurement noise is $\boldsymbol{\Sigma}$ and the design matrix $\mathbf{X}$ plays the role of $\mathbf{H}_m$. Using this data the prediction is refined to

$$\begin{aligned} \mathbf{P}_{m|m}^{-1} &= \mathbf{H}_m^{\dagger} \mathbf{R}_m^{-1} \mathbf{H}_m + \mathbf{P}_{m|m-1}^{-1} \\ \mathbf{x}_{m|m} &= \mathbf{P}_{m|m} (\mathbf{H}_m^{\dagger} \mathbf{R}_m^{-1} \mathbf{z}_k + \mathbf{P}_{m|m-1}^{-1} \mathbf{x}_{m|m-1}). \end{aligned} \tag{27}$$

With the above identifications these are the same equations as (23) and it is clear that the way BayesMGD predicts the optimal model parameters $\boldsymbol{\beta}$ is the same way that a Kalman filter predicts the system state $\mathbf{x}$.

4. Experimental comparison of optimisation algorithms

To compare the performance of BayesMGD, vanilla MGD and SPSA we ran VQE for different, well understood problem instances on real hardware with all three optimisation algorithms.

For BayesMGD, we used hyperparameters $A = 1$, $n_p = 1.5\dim(\boldsymbol{\beta})$, $\gamma = 0.3$, $\alpha = 0.602$, $\xi = 0.101$, $\delta = 0.6$ and $l = 0.2$, which gave consistently good results in simulations and were used in the experiments. We implemented MGD based on the description in Ref. 62. MGD has the same hyperparameters as BayesMGD, except for l, which is not a hyperparameter. In our experiments we used the same hyperparameter settings as for BayesMGD. $\boldsymbol{\beta}$ and $\boldsymbol{\Sigma}$ were initialised as

$$\begin{aligned} \boldsymbol{\beta}_0 &= 0 \\ \boldsymbol{\Sigma}_0 &= \mathrm{diag}(\underbrace{10^7}_{\Sigma_{\beta_0}}, \underbrace{10^7, \cdots, 10^7}_{\Sigma_{\beta_j}}, \underbrace{10^5, \cdots, 10^5}_{\Sigma_{\beta_{jk}}}), \end{aligned} \tag{28}$$

which has the effect that if the least squares fit is underdetermined the algorithm will first fit with a linear model and only set the β_{jk} to non-zero values if the data cannot be explained with a linear model.

Our implementation of SPSA was the same as that presented in Ref. 19, except that we only implemented a simple one-stage algorithm, not the three-stage approach used there, as we found that this was sufficient to obtain good performance. We used the same hyperparameters as that previous work, except that we reduced the stability constant A, as this was found to be more effective in experiments (we set $\alpha = 0.602$, $\gamma = 0.101$, $a = 0.2$, $c = 0.15$, $A = 1$).

As a figure of merit we show the energy difference between the exact energy expectation value one would obtain on a perfect device and the best exact energy attainable with the given VQE circuit. For a fair comparison, the number of shots per evaluation and number of evaluations per iteration was chosen such that the number of shots per iteration was the same for all optimisation runs shown in Supplementary Figure 7.

The left column of Supplementary Figure 7 shows that when the optimisation budget is given in shots instead of time, BayesMGD and MGD clearly outperform SPSA. However, when the optimisation budget is wall clock time, the rate of convergence of SPSA and (Bayes)MGD was roughly the same, as is best seen in the upper right panel of Supplementary Figure 7. In one of the SPSA runs on the 2×4 lattice the different sources of randomness in the SPSA algorithm conspired to send the parameters very close to the global minimum already

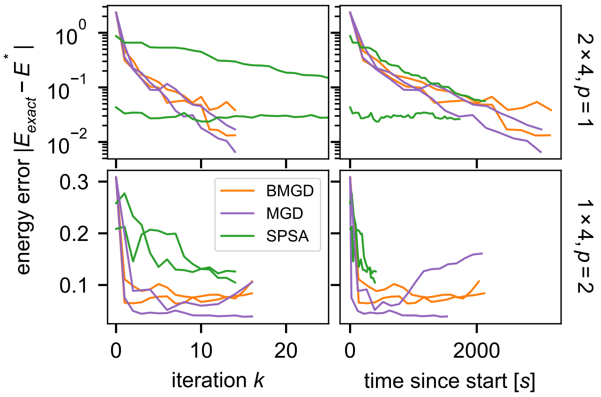

Supplementary Figure 7. Comparison of the B(ayes)MGD, MGD and SPSA optimisation algorithms. Shown is the energy difference between the classically evaluated, exact energies E_{exact} at the parameters produced at each step of the respective optimisation algorithm from noisy measurements on real hardware, and the best attainable energy at the given circuit depth E^*, as a function of the optimiser iteration (left column) and as a function of wall clock time since the start of the VQE run (right column). The upper row shows the results on a 2×4 lattice with ansatz depth 1 and the lower row shows the results on a 1×4 lattice with ansatz depth 2. Two runs are shown for each optimisation algorithm and lattice.

in the first iteration. These are the lower, orange, almost constant lines in the upper row and interestingly SPSA does not improve the parameters past that, while (Bayes)MGD was able to find better parameters even closer to the minimum. The lower row shows that, for harder instances with more free parameters (here 6 instead of 4), also (Bayes)MGD may fail to improve the parameters further once it got close to the minimum.

In the instances shown here, there was no clear difference between the performance of BayesMGD and MGD. However, in simulated tests of VQE for the antiferromagnetic Heisenberg model on the kagome lattice[63] with 12 qubits and 18 parameters we found that for $\eta := \frac{n_p}{(n_c+1)(n_c+2)/2} \geq 1$ the performance of BayesMGD and MGD is comparable, while for $\eta < 1$ BayesMGD often outperforms MGD. We recall that η is defined as the ratio between the number n_p of evaluation points taken in each iteration and the number $(n_c+1)(n_c+2)/2$ of evaluation points necessary for a fully determined quadratic fit. Hence $\eta < 1$ corresponds to an underdetermined quadratic fit, where good usage of prior information is especially important. These situations are shown in Supplementary Figure 8, where for $\eta = 0.7$ and 1.5 BayesMGD and MGD perform similar, while for $\eta = 0.05$ BayesMGD outperforms MGD. Note that in the given case for $\eta = 0.05$ the number of sample points is smaller than the number of parameters, i.e. even a linear fit is not fully specified.

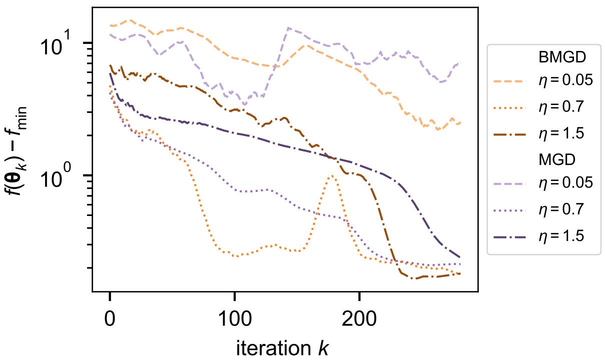

Supplementary Figure 8. Comparison of B(ayes)MGD and MGD with a simulated cost function with 18 parameters. As a cost function we chose the cost functions from Ref. 63 on 2×6 qubits with 3 layers. The number of shots per evaluation was scaled with $n_{\text{shots}} \sim \eta^{-1}$, making the number of shots per optimiser iteration constant. For clarity, we show only the exact function value at the current $\boldsymbol{\theta}$ instead of the noisy evaluations at the sample points. These are typically higher and far more spread out.

Supplementary Note 9 – Comparison with classical state of the art methods

In the main text we compared the results obtained by VQE with the best Slater determinant, showing that VQE outperforms this method. It is good to highlight that the Fermi-Hubbard model has been used as a test-bed for several classical approximation algorithms and these methods can be used to obtain approximations to ground state properties with better errors on larger systems that the ones studied here. For example, based on state of the art simulations of the Fermi-Hubbard model[64] using multireference projected Hartree-Fock (MRPFH) or unitary coupled cluster single double triple (UCCSDT), the error in the determination of the ground state energy per site between these methods is $\sim$ 0.03 (at $U = 8$ and close to half filling, on a system of 64 sites). In a more recent numerical study of superconductivity in the Fermi-Hubbard model[65], the gap in energy per site between two other leading methods (constrained-path (CP) auxiliary-field quantum Monte Carlo (AFQMC) and density matrix renormalization group (DMRG)) was $\sim$ 0.005, also for a system of 64 sites, with $U = 8$, close to half filling, and with no pairing field.

By comparison our measured VQE energy per site has an error $\sim$ 0.03 with respect to the perfect VQE ground state at depth 1, in the same regime of parameters but in a system 8x smaller. This hints that being able to increase the system size and number of VQE layers, while maintaining or somewhat improving the current level of error, could make VQE competitive with state of the art classical methods. We consider that outperforming the best Slater determinant at the size considered in this study, with a noisy device, is a necessary first goalpost to achieve.

From a complexity-theoretic point of view, approximating the ground state energy of a general quantum Hamiltonian is complete for the complexity class Quantum Merlin-Arthur (QMA)[66], which can be thought as the quantum version of the non-deterministic polynomial (NP) complexity class. As such QMA contains NP. In light of this, in general it is expected that not even a fully fault-tolerant quantum computer could approximate the energy of the most difficult instances without using exponential resources. Nevertheless, the expectation is that optimising over quantum circuits may offer yet another approximation technique not available with classical algorithms, and may be useful for physically meaningful instances. To determine beyond doubt if VQE is competitive against the plethora of approximate classical algorithms available, a thorough experimental analysis of VQE is needed in terms of the error scaling with system size for different parameters and regimes. It is known that classical approximations face different drawbacks as function of interaction strength, temperature or system size[64]. It is an open problem and an active area of research to fully characterise the power of VQE with increasing system size and varying physical conditions.

Supplementary References

1 J. Hubbard. Electron correlations in narrow energy bands. *Proc. R. Soc. Lond. A*, 276(1365):238–257, 1963.

2 A. Georges, G. Kotliar, W. Krauth, and M. J. Rozenberg. Dynamical mean-field theory of strongly correlated fermion systems and the limit of infinite dimensions. *Rev. Mod. Phys.*, 68:13–125, 1996.

3 D. P. Arovas, E. Berg, S. A. Kivelson, and S. Raghu. The Hubbard model. *Annu. Rev. Condens. Matter Phys.*, 13(1):239–274, 2022.

4 E. H. Lieb and F. Y. Wu. Absence of Mott transition in an exact solution of the short-range, one-band model in one dimension. *Phys. Rev. Lett.*, 20:1445–1448, 1968.

5 J. Carmelo and A. A. Ovchinnikov. The 1-d Hubbard model: A Landau Luttinger liquid. In A. A. Ovchinnikov and I. I. Ukrainskii, editors, *Electron-Electron Correlation Effects in Low-Dimensional Conductors and Superconductors*, pages 12–22. Springer, Berlin Heidelberg, 1991.

6 W. F. Brinkman and T. M. Rice. Application of Gutzwiller's variational method to the metal-insulator transition. *Phys. Rev. B*, 2:4302–4304, 1970.

7 B. S. Shastry, H. R. Krishnamurthy, and P. W. Anderson. Instability of the Nagaoka ferromagnetic state of the U=∞ Hubbard model. *Phys. Rev. B*, 41:2375–2379, 1990.

8 N. Linke, S. Johri, C. Figgatt, K. A. Landsman, A. Y. Matsuura, and C. Monroe. Measuring the Rényi entropy of a two-site Fermi-Hubbard model on a trapped ion quantum computer. *Phys. Rev. A*, 98:052334, 2018.

9 A. Montanaro and S. Stanisic. Compressed variational quantum eigensolver for the Fermi-Hubbard model, 2020. `arXiv:2006.01179`.

[10] P. Suchsland, P. K. Barkoutsos, I. Tavernelli, M. H. Fischer, and T. Neupert. Simulating a ring-like hubbard system with a quantum computer. *Phys. Rev. Research*, 4:013165, 2022.

[11] B. T. Gard and A. M. Meier. Classically efficient quantum scalable fermi-hubbard benchmark. *Phys. Rev. A*, 105:042602, 2022.

[12] F. Arute, K. Arya, R. Babbush, D. Bacon, J. C. Bardin, R. Barends, A. Bengtsson, S. Boixo, M. Broughton, B. B. Buckley, D. A. Buell, B. Burkett, N. Bushnell, Y. Chen, Z. Chen, Y.-A. Chen, B. Chiaro, R. Collins, S. J. Cotton, W. Courtney, S. Demura, A. Derk, A. Dunsworth, D. Eppens, T. Eckl, C. Erickson, E. Farhi, A. Fowler, B. Foxen, C. Gidney, M. Giustina, R. Graff, J. A. Gross, S. Habegger, M. P. Harrigan, A. Ho, S. Hong, T. Huang, W. Huggins, L. B. Ioffe, S. V. Isakov, E. Jeffrey, Z. Jiang, C. Jones, D. Kafri, K. Kechedzhi, J. Kelly, S. Kim, P. V. Klimov, A. N. Korotkov, F. Kostritsa, D. Landhuis, P. Laptev, M. Lindmark, E. Lucero, M. Marthaler, O. Martin, J. M. Martinis, A. Marusczyk, S. McArdle, J. R. McClean, T. McCourt, M. McEwen, A. Megrant, C. Mejuto-Zaera, X. Mi, M. Mohseni, W. Mruczkiewicz, J. Mutus, O. Naaman, M. Neeley, C. Neill, H. Neven, M. Newman, M. Y. Niu, T. E. O'Brien, E. Ostby, B. Pató, A. Petukhov, H. Putterman, C. Quintana, J.-M. Reiner, P. Roushan, N. C. Rubin, D. Sank, K. J. Satzinger, V. Smelyanskiy, D. Strain, K. J. Sung, P. Schmitteckert, M. Szalay, N. M. Tubman, A. Vainsencher, T. White, N. Vogt, Z. J. Yao, P. Yeh, A. Zalcman, and S. Zanker. Observation of separated dynamics of charge and spin in the Fermi-Hubbard model, 2020. `arXiv:2010.07965`.

[13] F. Arute, K. Arya, R. Babbush, D. Bacon, J. C. Bardin, R. Barends, S. Boixo, M. Broughton, B. B. Buckley, D. A. Buell, B. Burkett, N. Bushnell, Y. Chen, Z. Chen, B. Chiaro, R. Collins, W. Courtney, S. Demura, A. Dunsworth, E. Farhi, A. Fowler, B. Foxen, C. Gidney, M. Giustina, R. Graff, S. Habegger, M. P. Harrigan, A. Ho, S. Hong, T. Huang, W. J. Huggins, L. Ioffe, S. V. Isakov, E. Jeffrey, Z. Jiang, C. Jones, D. Kafri, K. Kechedzhi, J. Kelly, S. Kim, P. V. Klimov, A. Korotkov, F. Kostritsa, D. Landhuis, P. Laptev, M. Lindmark, E. Lucero, O. Martin, J. M. Martinis, J. R. McClean, M. McEwen, A. Megrant, X. Mi, M. Mohseni, W. Mruczkiewicz, J. Mutus, O. Naaman, M. Neeley, C. Neill, H. Neven, M. Y. Niu, T. E. O'Brien, E. Ostby, A. Petukhov, H. Putterman, C. Quintana, P. Roushan, N. C. Rubin, D. Sank, K. J. Satzinger, V. Smelyanskiy, D. Strain, K. J. Sung, M. Szalay, T. Y. Takeshita, A. Vainsencher, T. White, N. Wiebe, Z. J. Yao, P. Yeh, and A. Zalcman. Hartree-Fock on a superconducting qubit quantum computer. *Science*, 369(6507):1084–1089, 2020.

[14] A. Kandala, K. Temme, A. D. Córcoles, A. Mezzacapo, J. M. Chow, and J. M. Gambetta. Error mitigation extends the computational reach of a noisy quantum processor. *Nature*, 567(7749):491–495, 2019.

[15] S. E. Smart and D. A. Mazziotti. Quantum-classical hybrid algorithm using an error-mitigating n-representability condition to compute the Mott metal-insulator transition. *Phys. Rev. A*, 100:022517, 2019.

[16] C. Hempel, C. Maier, J. Romero, J. McClean, T. Monz, H. Shen, P. Jurcevic, B. P. Lanyon, P. Love, R. Babbush, A. Aspuru-Guzik, R. Blatt, and C. F. Roos. Quantum chemistry calculations on a trapped-ion quantum simulator. *Phys. Rev. X*, 8:031022, 2018.

[17] E. Farhi, J. Goldstone, and S. Gutmann. A quantum approximate optimization algorithm, 2014. `arXiv:1411.4028`.

[18] M. P. Harrigan, K. J. Sung, M. Neeley, K. J. Satzinger, F. Arute, K. Arya, J. Atalaya, J. C. Bardin, R. Barends, S. Boixo, M. Broughton, B. B. Buckley, D. A. Buell, B. Burkett, N. Bushnell, Y. Chen, Z. Chen, B. Chiaro, R. Collins, W. Courtney, S. Demura, A. Dunsworth, D. Eppens, A. Fowler, B. Foxen, C. Gidney, M. Giustina, R. Graff, S. Habegger, A. Ho, S. Hong, T. Huang, L. B. Ioffe, S. V. Isakov, E. Jeffrey, Z. Jiang, C. Jones, D. Kafri, K. Kechedzhi, J. Kelly, S. Kim, P. V. Klimov, A. N. Korotkov, F. Kostritsa, D. Landhuis, P. Laptev, M. Lindmark, M. Leib, O. Martin, J. M. Martinis, J. R. McClean, M. McEwen, A. Megrant, X. Mi, M. Mohseni, W. Mruczkiewicz, J. Mutus, O. Naaman, C. Neill, F. Neukart, M. Y. Niu, T. E. O'Brien, B. O'Gorman, E. Ostby, A. Petukhov, H. Putterman, C. Quintana, P. Roushan, N. C. Rubin, D. Sank, A. Skolik, V. Smelyanskiy, D. Strain, M. Streif, M. Szalay, A. Vainsencher, T. White, Z. J. Yao, P. Yeh, A. Zalcman, L. Zhou, H. Neven, D. Bacon, E. Lucero, E. Farhi, and R. Babbush. Quantum approximate optimization of non-planar graph problems on a planar superconducting processor. *Nat. Phys.*, 17(3):332–336, 2021.

[19] C. Cade, L. Mineh, A. Montanaro, and S. Stanisic. Strategies for solving the Fermi-Hubbard model on near-term quantum computers. *Phys. Rev. B*, 102:235122, 2020.

[20] B. A. Martin, P. Simon, and M. J. Rančić. Variational Hamiltonian Ansatz for 1d Hubbard chains in a broad range of parameter values, 2021. `arXiv:2111.11996`.

[21] T. Esslinger. Fermi-hubbard physics with atoms in an optical lattice. *Annu. Rev. Condens. Matter Phys.*, 1(1):129–152, 2010.

[22] J. Salfi, J. A. Mol, R. Rahman, G. Klimeck, M. Y. Simmons, L. C. L. Hollenberg, and S. Rogge. Quantum simulation of the Hubbard model with dopant atoms in silicon. *Nat. Commun.*, 7(1):11342, 2016.

[23] T. Hensgens, T. Fujita, L. Janssen, X. Li, C. J. Van Diepen, C. Reichl, W. Wegscheider, S. Das Sarma, and L. M. K. Vandersypen. Quantum simulation of a fermi–hubbard model using a semiconductor quantum dot array. *Nature*, 548(7665):70–73, 2017.

[24] L. Tarruell and L. Sanchez-Palencia. Quantum simulation of the Hubbard model with ultracold fermions in optical lattices. *C. R. Phys.*, 19(6):365–393, 2018.

[25] C. Gross and I. Bloch. Quantum simulations with ultracold atoms in optical lattices. *Science*, 357(6355):995–1001, 2017.

[26] A. Bohrdt, L. Homeier, C. Reinmoser, E. Demler, and F. Grusdt. Exploration of doped quantum magnets with ultracold atoms. *Ann. Phys. (N. Y.)*, 435:168651, 2021.

[27] C. Gross and W. S. Bakr. Quantum gas microscopy for single atom and spin detection. *Nat. Phys.*, 17(12):1316–1323, 2021.

[28] T. A. Hilker, G. Salomon, F. Grusdt, A. Omran, M. Boll, E. Demler, I. Bloch, and C. Gross. Revealing hidden antiferromagnetic correlations in doped Hubbard chains via string correlators. *Science*, 357(6350):484–487, 2017.

[29] G. Salomon, J. Koepsell, J. Vijayan, T. A. Hilker, J. Nespolo, L. Pollet, I. Bloch, and C. Gross. Direct observation of incommensurate magnetism in Hubbard chains. *Nature*, 565(7737):56–60, 2019.

[30] A. Mazurenko, C. S. Chiu, G. Ji, M. F. Parsons,

M. Kanász-Nagy, R. Schmidt, F. Grusdt, E. Demler, D. Greif, and M. Greiner. A cold-atom Fermi–Hubbard antiferromagnet. *Nature*, 545(7655):462–466, 2017.

[31] J. Koepsell, D. Bourgund, P. Sompet, S. Hirthe, A. Bohrdt, Y. Wang, F. Grusdt, E. Demler, G. Salomon, C. Gross, and I. Bloch. Microscopic evolution of doped mott insulators from polaronic metal to Fermi liquid. *Science*, 374(6563):82–86, 2021.

[32] P. T. Brown, D. Mitra, E. Guardado-Sanchez, R. Nourafkan, A. Reymbaut, C.-D. Hébert, S. Bergeron, A.-M. S. Tremblay, J. Kokalj, D. A. Huse, P. Schauß, and W. S. Bakr. Bad metallic transport in a cold atom fermi-hubbard system. *Science*, 363(6425):379–382, 2019.

[33] M. A. Nichols, L. W. Cheuk, M. Okan, T. R. Hartke, E. Mendez, T. Senthil, E. Khatami, H. Zhang, and M. W. Zwierlein. Spin transport in a mott insulator of ultracold fermions. *Science*, 363(6425):383–387, 2019.

[34] Z. Jiang, K. J. Sung, K. Kechedzhi, V. N. Smelyanskiy, and S. Boixo. Quantum algorithms to simulate many-body physics of correlated fermions. *Phys. Rev. Applied*, 9:044036, 2018.

[35] Z. Cai. Resource estimation for quantum variational simulations of the Hubbard model. *Phys. Rev. Applied*, 14:014059, 2020.

[36] I. D. Kivlichan, J. McClean, N. Wiebe, C. Gidney, A. Aspuru-Guzik, G. K.-L. Chan, and R. Babbush. Quantum simulation of electronic structure with linear depth and connectivity. *Phys. Rev. Lett.*, 120:110501, 2018.

[37] T. Shi, E. Demler, and J. Ignacio Cirac. Variational study of fermionic and bosonic systems with non-gaussian states: Theory and applications. *Ann. Phys. (N.Y.)*, 390:245–302, 2018.

[38] M. P. Kaicher, S. B. Jäger, and F. K. Wilhelm. Algorithm for initializing a generalized fermionic gaussian state on a quantum computer. *J. Phys. A*, 54(39):395303, Sep 2021.

[39] S. Endo, Z. Cai, S. C. Benjamin, and X. Yuan. Hybrid quantum-classical algorithms and quantum error mitigation. *J. Phys. Soc. Jpn.*, 90(3):032001, 2021.

[40] F. Arute, K. Arya, R. Babbush, D. Bacon, J. C. Bardin, R. Barends, R. Biswas, S. Boixo, F. G. S. L. Brandao, D. A. Buell, B. Burkett, Y. Chen, Z. Chen, B. Chiaro, R. Collins, W. Courtney, A. Dunsworth, E. Farhi, B. Foxen, A. Fowler, C. Gidney, M. Giustina, R. Graff, K. Guerin, S. Habegger, M. P. Harrigan, M. J. Hartmann, A. Ho, M. Hoffmann, T. Huang, T. S. Humble, S. V. Isakov, E. Jeffrey, Z. Jiang, D. Kafri, K. Kechedzhi, J. Kelly, P. V. Klimov, S. Knysh, A. Korotkov, F. Kostritsa, D. Landhuis, M. Lindmark, E. Lucero, D. Lyakh, S. Mandrà, J. R. McClean, M. McEwen, A. Megrant, X. Mi, K. Michielsen, M. Mohseni, J. Mutus, O. Naaman, M. Neeley, C. Neill, M. Y. Niu, E. Ostby, A. Petukhov, J. C. Platt, C. Quintana, E. G. Rieffel, P. Roushan, N. C. Rubin, D. Sank, K. J. Satzinger, V. Smelyanskiy, K. J. Sung, M. D. Trevithick, A. Vainsencher, B. Villalonga, T. White, Z. J. Yao, P. Yeh, A. Zalcman, H. Neven, and J. M. Martinis. Quantum supremacy using a programmable superconducting processor. *Nature*, 574(7779):505–510, 2019.

[41] R. Freeman. *Spin Choreography: Basic Steps in High Resolution NMR*. Oxford University Press, New York, 1998.

[42] K. J. Satzinger, Y. Liu, A. Smith, C. Knapp, M. Newman, C. Jones, Z. Chen, C. Quintana, X. Mi, A. Dunsworth, C. Gidney, I. Aleiner, F. Arute, K. Arya, J. Atalaya, R. Babbush, J. C. Bardin, R. Barends, J. Basso, A. Bengtsson, A. Bilmes, M. Broughton, B. B. Buckley, D. A. Buell, B. Burkett, N. Bushnell, B. Chiaro, R. Collins, W. Courtney, S. Demura, A. R. Derk, D. Eppens, C. Erickson, E. Farhi, L. Foaro, A. G. Fowler, B. Foxen, M. Giustina, A. Greene, J. A. Gross, M. P. Harrigan, S. D. Harrington, J. Hilton, S. Hong, T. Huang, W. J. Huggins, L. B. Ioffe, S. V. Isakov, E. Jeffrey, Z. Jiang, D. Kafri, K. Kechedzhi, T. Khattar, S. Kim, P. V. Klimov, A. N. Korotkov, F. Kostritsa, D. Landhuis, P. Laptev, A. Locharla, E. Lucero, O. Martin, J. R. McClean, M. McEwen, K. C. Miao, M. Mohseni, S. Montazeri, W. Mruczkiewicz, J. Mutus, O. Naaman, M. Neeley, C. Neill, M. Y. Niu, T. E. O'Brien, A. Opremcak, B. Pató, A. Petukhov, N. C. Rubin, D. Sank, V. Shvarts, D. Strain, M. Szalay, B. Villalonga, T. C. White, Z. Yao, P. Yeh, J. Yoo, A. Zalcman, H. Neven, S. Boixo, A. Megrant, Y. Chen, J. Kelly, V. Smelyanskiy, A. Kitaev, M. Knap, F. Pollmann, and P. Roushan. Realizing topologically ordered states on a quantum processor, 2021. `arXiv:2104.01180`.

[43] L. Viola and S. Lloyd. Dynamical suppression of decoherence in two-state quantum systems. *Phys. Rev. A*, 58:2733–2744, 1998.

[44] B. Pokharel, N. Anand, B. Fortman, and D. A. Lidar. Demonstration of fidelity improvement using dynamical decoupling with superconducting qubits. *Phys. Rev. Lett.*, 121:220502, 2018.

[45] Technically, this is a consequence of time-reversal symmetry combined with spin-flip symmetry.

[46] S. Endo, S. Benjamin, and Y. Li. Practical quantum error mitigation for near-future applications. *Phys. Rev. X*, 8:031027, 2018.

[47] A. Kandala, A. Mezzacapo, K. Temme, M. Takita, M. Brink, J. M. Chow, and J. M. Gambetta. Hardware-efficient variational quantum eigensolver for small molecules and quantum magnets. *Nature*, 549(7671):242–246, 2017.

[48] F. B. Maciejewski, Z. Zimborás, and M. Oszmaniec. Mitigation of readout noise in near-term quantum devices by classical post-processing based on detector tomography. *Quantum*, 4:257, 2020.

[49] Y. Chen, M. Farahzad, S. Yoo, and T.-C. Wei. Detector tomography on IBM quantum computers and mitigation of an imperfect measurement. *Phys. Rev. A*, 100(5):052315, 2019.

[50] A. Montanaro and S. Stanisic. Error mitigation by training with fermionic linear optics, 2021. `arXiv:2102.02120`.

[51] P. Czarnik, A. Arrasmith, P. J. Coles, and L. Cincio. Error mitigation with Clifford quantum-circuit data. *Quantum*, 5:592, 2021.

[52] B. M. Terhal and D. P. DiVincenzo. Classical simulation of noninteracting-fermion quantum circuits. *Phys. Rev. A*, 65:032325, 2002.

[53] D. Wecker, M. B. Hastings, and M. Troyer. Progress towards practical quantum variational algorithms. *Phys. Rev. A*, 92:042303, 2015.

[54] H. Theil. A rank-invariant method of linear and polynomial regression analysis (parts 1-3). *Ned. Akad. Wetensch. Proc. Ser. A*, 53:386–392, 1950.

[55] P. K. Sen. Estimates of the regression coefficient based on Kendall's tau. *J. Am. Stat. Assoc.*, 63:1379–1389, 1968.

[56] W. J. Huggins, S. McArdle, T. E. O'Brien, J. Lee, N. C. Rubin, S. Boixo, K. B. Whaley, R. Babbush, and J. R. McClean. Virtual distillation for quantum error mitigation. *Phys. Rev. X*, 11:041036, 2021.

[57] B. Koczor. Exponential error suppression for near-term

quantum devices. *Phys. Rev. X*, 11:031057, 2021.

58 L. P. Kadanoff. More is the same; phase transitions and mean field theories. *J. Stat. Phys.*, 137(5):777, 2009.

59 E. Marciniak and J. Wesolowski. Asymptotic Eulerian expansions for binomial and negative binomial reciprocals. *Proc. Am. Math. Soc*, 127(11):3329–3338, 1999.

60 R. E. Kalman. A new approach to linear filtering and prediction problems. *J. Basic Eng.*, 82(1):35–45, 03 1960.

61 D. G. Hoag. Apollo guidance, navigation and control. Technical Report E-2411, MIT instrumentation laboratory, Cambridge 39, Massachusetts, 4 1969.

62 K. J. Sung, J. Yao, M. P. Harrigan, N. C. Rubin, Z. Jiang, L. Lin, R. Babbush, and J. R. McClean. Using models to improve optimizers for variational quantum algorithms. *Quantum Sci. Technol.*, 5(4):044008, 2020.

63 J. L. Bosse and A. Montanaro. Probing ground-state properties of the kagome antiferromagnetic heisenberg model using the variational quantum eigensolver. *Phys. Rev. B*, 105:094409, Mar 2022.

64 J. P. F. LeBlanc, A. E. Antipov, F. Becca, I. W. Bulik, G. K.-L. Chan, C.-M. Chung, Y. Deng, M. Ferrero, T. M. Henderson, C. A. Jiménez-Hoyos, E. Kozik, X.-W. Liu, A. J. Millis, N. V. Prokof'ev, M. Qin, G. E. Scuseria, H. Shi, B. V. Svistunov, L. F. Tocchio, I. S. Tupitsyn, S. R. White, S. Zhang, B.-X. Zheng, Z. Zhu, and E. Gull. Solutions of the two-dimensional Hubbard model: Benchmarks and results from a wide range of numerical algorithms. *Phys. Rev. X*, 5:041041, 2015.

65 M. Qin, C.-M. Chung, H. Shi, E. Vitali, C. Hubig, U. Schollwöck, S. R. White, and S. Zhang. Absence of superconductivity in the pure two-dimensional hubbard model. *Phys. Rev. X*, 10:031016, 2020.

66 D. Aharonov and T. Naveh. Quantum NP - A survey, 2002. `arXiv:0210.077`.
